# Fermentative Lactic Acid Production From Lignocellulosic Feedstocks: From Source to Purified Product

**DOI:** 10.3389/fchem.2022.823005

**Published:** 2022-03-04

**Authors:** Dragomir Yankov

**Affiliations:** Chemical and Biochemical Reactors Laboratory, Institute of Chemical Engineering, Bulgarian Academy of Sciences, Sofia, Bulgaria

**Keywords:** lignocellulosic biomass, lactic acid, pretreatment, fermentation, separation

## Abstract

The second (lignocellulosic biomass and industrial wastes) and third (algal biomass) generation feedstocks gained substantial interest as a source of various value-added chemicals, produced by fermentation. Lactic acid is a valuable platform chemical with both traditional and newer applications in many industries. The successful fractionation, separation, and hydrolysis of lignocellulosic biomass result in sugars’ rich raw material for lactic acid fermentation. This review paper aims to summarize the investigations and progress in the last 5 years in lactic acid production from inexpensive and renewable resources. Different aspects are discussed—the type of raw materials, pretreatment and detoxification methods, lactic acid-producers (bacteria, fungi, and yeasts), use of genetically manipulated microorganisms, separation techniques, different approaches of process organization, as well as main challenges, and possible solutions for process optimization.

## 1 Introduction

The intensive economic growth during the past century is accompanied by high energy consumption. Nowadays fossil fuels (coil, petroleum, and natural gas) are still the main energy source and raw material for the production of various chemicals. The fossil fuels were formed and stored underground for millions of years and their extensive use has led to the situation where the present vegetation on Earth cannot treat the emitted carbon dioxide by photosynthesis (Damyanova and Beschkov 2020). As a consequence, the strong emissions of carbon dioxide and greenhouse gases affect and change the climate. One of the ways to cope with this global problem is to close the natural carbon cycle using renewable sources as a platform for biofuels and chemicals production, and thus enabling recycling of the biological sources and consumption of the resulting carbon dioxide by photosynthesis (Beschkov et al., 2020).

A major step in the development of a sustainable, industrial society will be the shift from dependence on oil to the use of renewable resources. In the prospect of environmental sustainability, the utilization of agro-industrial waste residues as feedstock for the production of both biofuels and basic synthetic chemicals in biorefineries has gained widespread attention (Yaashikaa et al., 2022). In the long run, oil use can be eliminated and gas emissions reduced. The market share of biotechnological processes for the production of various chemical products is expected to increase in the coming years from 5 to 20%. Organic acids are a key group of chemicals that can be produced microbiologically. Functional groups that need to be introduced through an expensive multistage oil oxidation process are present in plant materials such as carbohydrates.

Cellulosic biomass is an abundant and sustainable source of valuable chemicals that are truly unique for making various organic products. It includes agricultural wastes (corn stover, spent grains, and sugarcane bagasse), some municipal solid wastes (waste paper), wastes from forestry residues (mill wastes and sawdust), herbaceous (switchgrass), and woody (poplar trees) crops. Such materials are plentiful and accessible in many regions of the world and can be competitive in price with petroleum and thus opening up a new route to manufacturing organic fuels and chemicals. Despite the high availability, the degradation of biomass is a substantial challenge. Hence, it is necessary to integrate several unit processes such as biochemical, thermochemical, physical, and catalytic conversion to produce a wide range of bio-based products (Velvizhi et al., 2022).

Lgnocellulosic biomass is composed of cellulose linear chains formed from glucose units. Cellulose fibers are held together with hemicellulose (branched heteropolymer formed by various hexoses and pentoses) and lignin (complex heteropolymer built from phenylpropanoid units). The specific structure of lignocellulose restricts the access of enzymes to cellulose and hemicellulose fibers and hinders effective hydrolysis. In view to converting lignocellulose to valuable chemicals by fermentation, this structure must be destructed by some pretreatment. The purpose of the pretreatment is to decrease the crystallinity of cellulose and degree of polymerization, destruct hydrogen bonds between fibers, and thus increase the area accessible for the enzymes’ action. The lignin should be at least partially removed and cellulose and hemicellulose efficiently hydrolyzed to fermentable sugars. Various pretreatment techniques have been used with the goal of breakdown the lignocellulosic complex and liberating sugars for further use.

Lignocellulosic biomass of various sources can be used for the production of different chemicals such as fuels (ethanol, methane, hydrogen), enzymes (cellulases, amylase, protease), high-value chemicals (hydroxycinnamic acid, lactic acid, xylitol), etc.

The present review aims to summarize in-depth the latest achievements in the field of fermentative lactic acid production from renewable sources –various substrates, microorganisms including gene manipulated, as well as process organization and downstream techniques.

## 2 Lactic Acid

**FIGURE 1:**
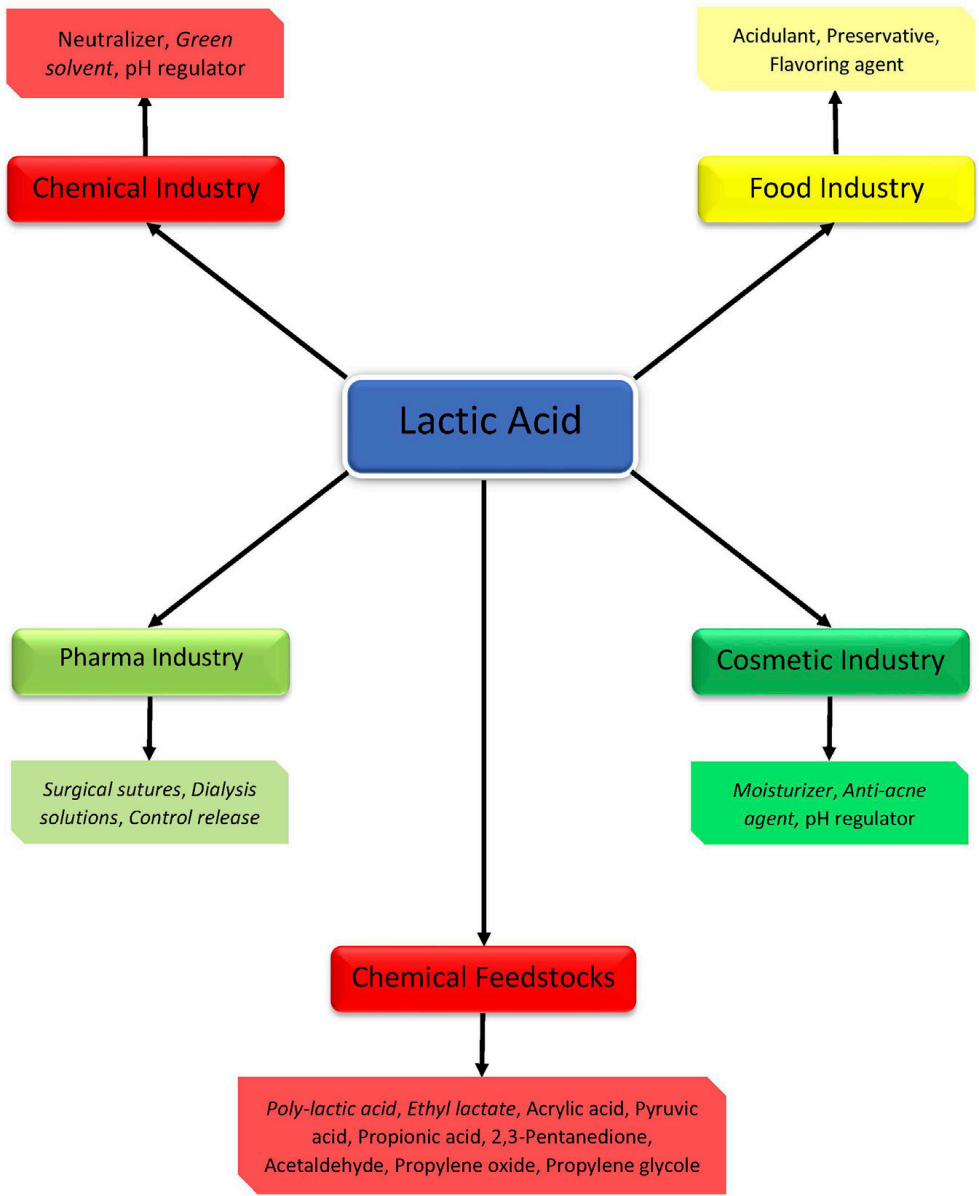
Applications of lactic acid in different industries (Newer application are given in italic).

Lactic (2-hydroxypropanoic) acid (LA) is a valuable platform chemical with both traditional and newer applications (Figure 1). It was widely used as a neutralizer, preservative, or acidulant in food and beverage, cosmetic, pharmaceutical, and other industries. Recently, lactic acid is applied as the building block for various biodegradable polymers or precursor for environmentally friendly solvents. The annual global market of lactic acid in 2020 is valued at USD 1.1 billion with an increasing tendency to double till 2025 (Lactic Acid Market by Application (Biodegradable Polymers, Food & Beverages, Pharmaceutical Products) 2020). The global market for lactic acid was 750.00 kilotons in 2017 and is projected to reach 1,845.00 kilotons by 2022 (Global Lactic Acid Market 2017-2025 - Growth Trends, Key Players, Competitive Strategies and Forecasts - Research and Market 2017).

**FIGURE 2:**
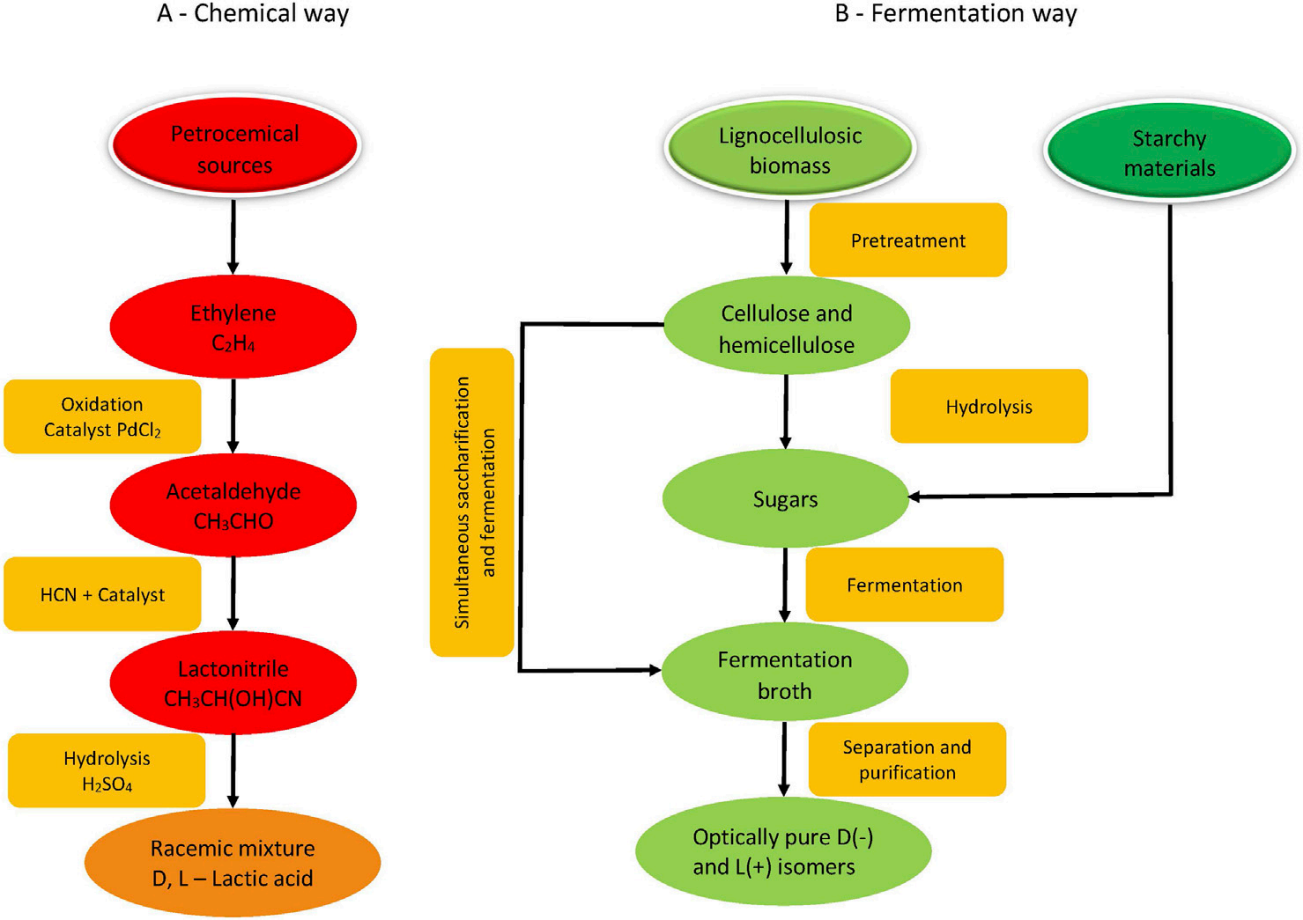
Methods for lactic acid production (A-Chemical way; B—Fermentation way).

Nowadays, lactic acid is produced by chemical or fermentation way. In the chemical route (Figure 2A), lactic acid is produced from petrochemical sources by a multistep reaction scheme. It includes catalytic oxidation of ethylene, conversion of obtained acetaldehyde to lactonytrile end its hydrolysis to a racemic mixture of D (-) and L (+) lactic acid. Of course, there are other methods for the chemical synthesis of lactic acid like oxidation of propylene or propylene glycol, hydrolysis of chloropropionic acid, but they are not economically and technically feasible (Krishna et al., 2018). Fermentative production of lactic acid is based on the conversion of different sugars (glucose, lactose), starchy or lignocellulosic hydrolysates by different microorganisms, mainly lactobacilli (Figure 2B). Factors affecting lactic acid fermentation (pH, temperature, nutrients, substrate, product concentrations, etc.) are discussed in the review of Rawoof et al. (Rawoof et al., 2021).

- *pH*—for most lactobacilli the optimum value for effective lactic acid production is between 5 and 7. Unfortunately, accumulation of the lactic acid leads to decreasing pH, even outside the optimum value, leading to a decrease in microorganisms’ growth and lactic acid production. Additionally, high lactic acid concentration inhibits cellular metabolism. A decrease in pH could be overcome by neutralization with strong bases like sodium, ammonium, or calcium hydroxide, or low molecular mass water-soluble amines. Another way is *in-situ* product removal during fermentation. Discovering or engineering the acid-tolerant strains is a challenge. This will permit to decrease the product inhibition, and overall cost of the process, eliminating the use of neutralizers.
- *Temperature*—the temperature is an important factor for bacterial growth and lactic acid production. Most of the lactobacilli are mesophilic (25–40°C). An increase in the operational temperature will result in decreasing contamination and increasing substrate hydrolysis. Very often, the optimal temperature for microbial growth differs from that for maximal lactic acid production. The selection of strains capable to grow at thermophilic (40–65°C) or extreme thermophilic (65–80°C) conditions will be very favorable.
- *Media composition*—the complexity of the fermentation media (carbon and nitrogen sources, minerals, vitamins) is a factor increasing the cost of lactic acid production. Carbon is an indispensable element and the replacement of costly individual sugars with cheap agricultural and industrial wastes is a step towards decreasing the total cost. Nitrogen is a major component in the anabolic and catabolic processes. Usually, complex nitrogen sources like peptone, yeast extract, and meat extract are used. Alternatives for cheap nitrogen sources are corn steep liquor, hydrolysate of fish waste, and wheat bran extract. DDGS (about 30% protein) is an attractive alternative because its hydrolysis results in carbon sources and nitrogen at a time. The ratio between carbon and nitrogen (C/N) also is very important. An optimal C/N ratio results in a positive effect on lactic acid fermentation. Usually, the optimal C/N ratio is between 3 and 7 (Gómez-Gómez et al., 2015), (Gilver Rosero Chasoy et al., 2020), (Mejia-Gomez and Balcazar 2020).
- *Sugars concentration*—at a high initial substrate concentration some problems such as longer lag phase, decreased activity, cellular lysis, and osmotic stress could occur, resulting in reduced substrate consumption and low lactic acid production. The effect of substrate inhibition can be diminished by adding osmo-protective substances, fed-batch fermentation mode, developing high sugars tolerant strains. Substrates originating from lignocellulosic sources usually are a mix of pentoses and hexoses. Lactic acid bacteria possess a hierarchical pattern of sugar utilization—glucose is utilized first and then other sugars. This leads to carbon catabolite repression, a decrease in fermentation efficiency, and low product concentration. The problem can be solved by using pentoses and hexoses utilizing strains in co-fermentation mode. Another way is isolating or engineering strains with increased carbon catabolic repression resistance.
- *Effect of end- and by-product accumulation—*the effect of product accumulation and subsequences were discussed above. Glucose and xylose are the main conctituents of agrocellulosic hydrolisates. While glucose undergoes conversion to lactic acid mainly by homo-fermentation, xylose is converted by the hetero-fermentative pathway, leading to the formation of by-products like ethanol and acetic acid. The accumulation of by-products leads to a decrease in lactic acid yield and an increase in the cost of product recovery. Again, the selection of novel strains and genetically modifying the existing strains to follow the desired pathway is the possible solution to the problem.
- *Sensitivity to toxic compounds*—most of the pretreatment methods lead to the formation of inhibitory compounds—furfural, 5-hydroxymethyl furfural, levulinic acid, formic acid, *p*-coumaric acid, ferulic acid, etc. These compounds exhibit an inhibitory effect on the enzyme’s action, cellular growth, and lactic acid production. Different approaches can be used for elimination of inhibitory effect—use of pretreatment methods leading to less liberation of inhibitors, chemical or biological neutralization of the inhibitors, use of adaptive evaluated, inhibitor-tolerant, or engineered strains.

Some bottlenecks hinder effective lactic acid production. The major one is product inhibition. Accumulation of the lactic acid leads to a fast drop in the medium pH, outside of the optimum value for the producer strain. This results in low substrate conversion and low final product concentration. The problem may be overcome either by *in situ* product removal or by using acid-tolerant bacteria. The cost of raw material and nutritional additives are with the major contribution to the overall cost of lactic acid. Usually, the pure sugars used as a substrate for lactic acid fermentation are very costly and take part in the human food chain. According to S. Tejayadi and M. Cheryan in the case of lactic acid production from whey permeate, the cost of whey (including transportation) was 35% and for yeast extract—38% of the total cost (Tejayadi and Cheryan 2005). Akerberg and Zacchi reported that the major operational costs (raw material, saccharification, fermentation, and electrodialysis) contributed about 80% to the total cost in case of lactic acid production from wheat flour (Akerberg and Zacchi 2000). For using starch or other natural polymers, like inulin, it is necessary either to hydrolyze it to individual sugars or use lactic acid-producing bacteria with relevant enzyme activity. In recent years, a new process for lactic acid production attract the interest of researchers in the field—catalytic conversion of lignocellulosic biomass. The process is led in the presence of homogeneous and heterogeneous catalysts at lower temperatures or under subcritical conditions. For example, lactic acid production from empty fruit palm oil bunch under hydrothermal conditions in the presence of metal salts was described (Sitompul et al., 2014) and (Chin et al., 2016). In an extensive and comprehensive review, Mäki-Arvela et al. (2014) describe the research since 2000 on the production of lactic acid from biomass and its transformation to commodity chemicals. The mechanisms in the production of lactic acid and its derivatives in the presence of homogeneous and heterogeneous catalysts were described, as well as reaction conditions, catalysts’ properties, stability, and reuse. The authors also discussed some reactor technologies and kinetic modeling of the processes in hydrogenation and esterification of lactic acid.

In any case, it is clear that finding a cheap, abundant, and easily accessible row material is a key factor for an effective and economically profitable method for fermentative lactic acid production. Lignocellulosic biomass especially forest biomass, waste, and inedible plant materials, as well as various agricultural crop residues, seems to be a promising substrate for an effective and economically profitable method for fermentative lactic acid production which non-competes with the human food chain. Recently, several review papers concerning lignocellulosic biomass utilization as a source for bio-based chemicals production have been published. (Tišma et al., 2021; Banu et al., 2021; Haldar and Purkait 2020; Usmani et al., 2021; Nwamba et al., 2021).

Regarding lactic acid production from lignocellulosic biomass, there are some reviews published in the last 2 years considering different aspects of the issue—microorganisms (Abedi and Hashemi 2020), sources (Lopez-Gomez et al., 2020; Ajala et al., 2020), pretreatment methods (Huang et al., 2021; Mankar et al., 2021), separation (De Oliveira et al., 2020; Li et al., 2021), techno-economic analysis (Li et al., 2021; Manandhar and Shah 2020; Daful and Görgen 2017), etc. Thygesen et al. (2021) summarized recent data on the valorization of municipal organic waste into purified lactic acid - pretreatment, enzymatic hydrolysis, LA fermentation, and downstream processing.

### 2.1 Problems and Obstacles in Fermentative Lactic Acid Production From Lignocellulosic Biomass

In every step of lactic acid production by fermentation from lignocellulosic feedstocks, from pretreatment to separation, there are various bottlenecks, which must be overcome for the realization of an effective and low-cost process.

Regardless of the undoubted advantages of using lignocellulosic biomass as raw material for lactic acid production, like low cost and global availability, main problems can be summarized as follows:

- in pretreatment step—-i) resistant nature of biomass and difficulties in full separation of lignin from cellulose and hemicellulose; ii) energy consumption, toxicity, and environmental problems of some used chemicals; iii) release of inhibitory compounds; iv) high cost of hydrolytic enzymes and their product inhibition.
- in fermentation step—i) nutritional requirements of lactic acid bacteria; ii) carbon catabolite repression; iii) product and substrate inhibition; iv) by-product formation in hetero-fermentation.
- in separation and purification step—i) low concentration of feeding stream; ii) more complex composition of fermentation broth in comparison with first-generation LA production; iii) high cost of downstream processes; iv) not all separation methods are tested on second-generation LA; v) ecological problems connected with classical precipitation separation.

More details for different problems and possible solutions are given in (Cubas-Cano et al., 2018; Tan et al., 2018; Grewal et al., 2020).

## 3 Lignocellulosic Feedstocks

**FIGURE 3:**
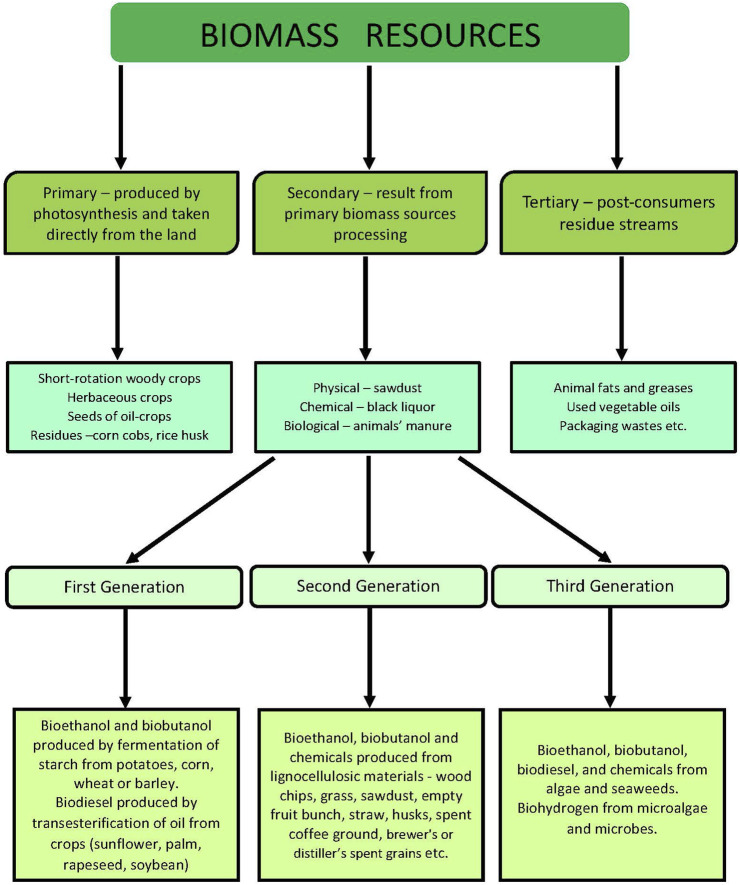
Classification of biomass resources.

**FIGURE 4:**
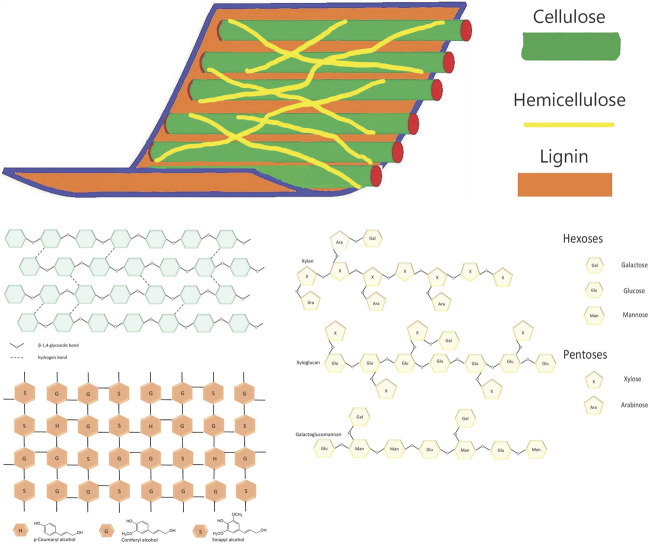
Sketch of lignocellulose structure.

The term biomass generally includes the mass of organic material in all living organisms on Earth (microorganisms, algae, plants, and animals). The main building elements of biomass are carbon, hydrogen, oxygen, and nitrogen, and similarly to fossil feedstocks, the biomass can be used for various chemicals production. Biomass is abundant and renewable and can be divided into three groups (Figure 3). Primary resources are produced directly by photosynthesis and taken from the land; secondary resources result from the processing of primary sources and tertiary—post-consumers residue streams. The greater part of plant biomass consists of lignocellulose—a complex of three polymers (cellulose, hemicellulose, and lignin) and a small number of extractives, pectin, and ash. A sketch of lignocellulose structure is given in Figure 4.

Cellulose is a linear polysaccharide comprising d-glucose units, linked by β (1–4)-glycoside bonds (in contrast to α (1–4) bonds in starch amylose). Cellulosic molecules are located in parallel, forming primary fibrils, stabilized by hydrogen bonds. The sections with a strictly parallel arrangement and additional hydrogen bonding, are referred to as crystalline, in contrast, to those in which such arrangement is missing. The latter are called amorphous. The cellulose fibrils can further interact to form cellulose fibers. The cellulose is very stable and insoluble in water, diluted acids, and the majority of common solvents, but is soluble in concentrated acids, and alkali bases (for DP smaller than 100).

Hemicellulose is a branched polymer built from different saccharides—pentoses (xylose, arabinose), hexoses (glucose, mannose, galactose) as well as some uronic acids (glucuronic, galacturonic acid). For example, the main components in softwood hemicelluloses are built from hexoses and those of hardwood from pentoses. The polymerization degree of hemicelluloses is smaller than in cellulose. Hemicellulosic polysaccharides are soluble in bases (10% KON or 18% NaOH), dimethylsulphoxide, and in rare cases in hot water.

Lignin is a highly branched amorphous phenolic polymer composed mainly of p-coumarin alcohol (H unit), coniferyl alcohol (G unit), and sinapyl alcohol (S unit). Lignin is a highly cross-linked polymer with long chains. The structure of lignin differs considerably from its source (type of plant, and even different parts of the same plant), for example, the lignin, derived from softwood is built predominantly of G units, hardwood lignin is mostly comprised of G and S units, while lignin from grass contains all three units.

Cellulose fibers are stacked together by hydrogen bonds with hemicellulose and pectin. Lignin interacts with cellulose and hemicellulose not only by hydrogen bonding but with covalent bonds and thus forming a rigid complex structure difficult for chemical or biochemical degradation.

Lignocellulosic biomass differs considerably depending on its origin. In general, the composition is cellulose 30–60%, hemicellulose 20–40%, and lignin 15–25% (Norrrahim et al., 2021). Reshmy et al. (2022) discussed the selection of lignocellulosic sources, advanced pretreatment methods for various types of cellulose for use in cost-effective bio-refineries for future industrial application. A short description of major platform chemicals and biomaterials as well as different biofuels such as biomethane, bioethanol, and biohydrogen, is given. In Table 1 data for different lignocellulosic feedstocks composition are summarized.

**TABLE 1:** Composition of different lignocellulosic feedstocks.

Lignocellulosic biomass	Cellulose%	Hemicellulose %	Lignin %	Reference
Softwood chips (pine)	46.7	23.5	28.1	Wu et al., (2020)
Softwood pellets (spruce, pine, and fir)	42.4	21.6	27.5	
Hardwood (aspen) chips	46	19	28	Wu et al. (2019)
Softwood (Lodgepole pine) chips	46.7	23.5	28.1	Wu et al., (2020)
Softwood (spruce, pine and fir) pellets	42.4		27.5	
*Sophora flavescens* root	46.6	19.5	20.2	Ma et al. (2020)
Sugar palm trunk	40.1	16.5	27.2	Erliana et al. (2020)
Pine sawdust	24.9	31.5	36.6	Rapado et al. (2021)
Oil Palm Empty Fruit Bunch	29.4	14.4	22.7	Aini et al. (2018)
Date palm wastes				
Leaves	59,1	16.7	16.1	Alrumman (2016)
Leaf bases	51.5	24.4	18.5	
Fibrous material	43.2	12.8	24.1	
Pulp mill residue	80.9	16.2	1.7	Moraes et al. (2016)
Orange peel waste	19.1	14.8	6.2	Bustamante et al. (2020)
Pressed recycled paper sludge	34.1	7.9	20.4	Marques et al. (2017)
Cassava bagasse	13.5	5.8	2.8	Chen et al. (2020)
Sugarcane bagasse	34.7	25.2	19.2	Wischral et al. (2019)
	47.4	9.7	5.4	Oliveira et al. (2019)
	41.4	32.4	8.3	Zheng et al. (2021)
	13.5	26.0	22.5	Chen et al. (2020)
	47.2	5.8	2.8	Baral et al. (2021)
	43.8	19.6	27.7	Grewal and Khare (2018)
	34.5	32.7	23.2	González-Leos et al. (2020)
	36.6	16.0	32.1	Nalawade et al. (2020)
	45.5	20.8	22.1	Nalawade et al. (2020)
	33.0	22.3	29.9	Li et al. (2021)
	39.6	26.2	23.4	Pin et al. (2019)
	27.9	25.6	2.8	Azaizeh et al. (2020)
Bagasse sulphite pulp	73.8	14.3	5.9	Zhou et al. (2016)
Sweet sorghum bagasse	38.5	23.4	21.4	Wang et al. (2016)
Banana peel	14.0	13.0	17.0	Martínez-Trujillo et al. (2020)
Banan peel	12.2	10.2	2.9	Redondo-Gómez et al. (2020)
Banana rachis	23.0	11.2	10.8	
Banana penduncle	35.8	20.7	6.16	Azaizeh et al. (2020)
Carob biomass	19.0	0.35	28.4	Azaizeh et al. (2020)
Brewers spent grains	16.8	28.4	27.8	Mussatto and Roberto (2006)
	21.7	19.3	19.4	Meneses et al. (2013)
	25.3	41.9	16.9	Silva et al. (2004)
	21.9	29.6	20.6	Carvalheiro et al. (2004)
Corn stover	41.2	30.1	19.4	Rahman et al. (2020)
	37.1	29.6	20.8	Zhang et al., 2020
	31.3	26.9	16.4	Sun et al. (2021)
	32.6	26.4	31.0	Cheng et al. (2018)
	31.2	22.3	20.8	Han et al. (2019)
	38.8	23.6	18.4	Ma et al. (2016)
	45.0	21.0	17.0	He et al. (2016)
	43.6	19.2	22.6	Zhang et al. (2021)
	33.0	26.9	20.8	Wei et al. (2018)
	40.1	15.1	18.3	Chen et al. (2020)
	34.7	21.3	21.7	Shi et al. (2021)
Corn cob	37.0	34.3	16.4	Lin et al. (2020)
	70.0	9.7	16.2	Alhafiz et al. (2020)
	36.7	30.0	23.3	Sánchez et al. (2012)
	32.6	31.7	16.9	He et al. (2017)
Wheat straw	41.1	37.5	13.5	Grewal and Khare (2018)
	40.5	26.1	18.1	Cubas-Cano et al. (2019)
Rye straw	38.0	25.5	27.1	Schroedter et al. (2021)
Digestate of energy corn silage	29.3	22.4	33.5	Schroedter et al. (2021)
Sorghum straw	25.0	27.5	20.2	Wu et al. (2021)
Rice straw	33.3	23.3	17.5	Yao et al. (2007)
	44.6	29.0	12.4	Jaichakan et al. (2021)
	32.2	18.9	24.0	Younas et al. (2016)
	34.2	17.2	21.4	Chen et al. (2019)
	35.0	18.0	15.0	Yadav et al. (2021)
	34.5	21.3	13.3	Tu et al. (2019)
Rice husk	47.6	19.1	19.3	Jaichakan et al. (2021)
Soybean hulls	35.8	23.1	9.1	Rojas et al. (2014)
Chestnut shell	27.6	15.7	27.5	He et al. (2016)
	28.1	16.7	23.2	He et al. (2016)
Pecan nutshell	28.7	8.8	27.1	Qin et al. (2017)
Deoiled cottonseed cake	24.4	14.3	5.2	Grewal and Khare (2018)
Spent coffee grounds	24.3	24.8	13.5	Girotto et al. (2018)
	10.8	28.3	10.7	Cruz-Lopes et al. (2017)
	12.4	39.1	23.9	Ballesteros et al. (2014)
	7.0	43.0	37.0	Koo et al. (2019)
Distilery stillage - Rye	16.8	29.6	15.6	Mikulski and Kłosowski (2018)
Wheat	18.6	34.1	9.5	
Corn	32.2	20.9	3.2	
Distillers spent grain -wheat	11.1	20.3	2.0	Zaini et al. (2019)

In what follows a brief data for the most used lignocellulosic feedstocks are given.

### 3.1 Corn Residues

The world corn production for 2020/2021 is estimated at around 1.125 billion metric tons (Shahbandeh, 2021a). It is estimated that for each bushel of shelled corn 50 pounds of corn residue (cobs, leaves, stalks, and husks) are also produced (McCutcheon and Samples 2002). It means that an enormous amount of corn residues are available for converting into value-added products.

### 3.2 Brewer’s Spent Grains

Brewer’s spent grains (BSG) are the major by-product in the brewing industry. The quantity of residue material in beer production is about 24.4 kg per 100 L of beer produced (about 85% of total generated by-products are brewer’s spent grains or approximately 20 kg per 100 L of beer (Reinold 1997)). The worldwide beer production in the last decade is around 1.93 billion hectoliters (Conway 2021).

### 3.3 Sugarcane Bagasse

Sugarcane is a perennial grass mainly used for sugar production. Annually around 1,89 billion tonnes of sugarcane are produced worldwide and 250–270 kg of sugarcane bagasse are generated from each ton of sugarcane (Wischral et al., 2019).

### 3.4 Spent Coffee Grounds

Coffee is surely one of the most popular beverages consumed in the world. The volume of world coffee production in 2019/2020 is estimated to be 163.7 million 60-kg bags (Shahbandeh, 2021b). Approximately half (in dry weight basses) of the coffee is separated in the form of spent coffee grounds during the preparation of coffee beverages or the manufacturing of instant coffee or instant coffee-making process (Kim et al., 2019). According to (Mussatto et al., 2011) 650 kg of Spent Coffee Grounds (SCG) are generated from each ton of green coffee.

### 3.5 Distillery’s Dried Grains With Solubles

Distillery’s Dried Grains With Solubles (DDGS) represent the most important by-product in bioethanol production. After ethanol distillation, the thin stillage from the fermentation broth was decanted, and the solid is dried to produce DDGS. The world bioethanol production (from sugarcane, wheat, corn, and other feedstocks) in 2020 was estimated at around 100 billion liters (Bio-ethanol market-growth, trends, covid-19 impact, and forecasts (2021 - 2026) 2021). The production of the first generation of ethanol is almost strictly differentiated by countries (mainly from corn in the United States and sugarcane in Brazil). Taking into account that from bushel (25.4 kg) grains about 8.2 kg of ethanol is produced, releasing about 7.7 kg of DDGS (Jacques et al., 2003) it is easy to calculate that about 73 million of tonnes DDGS are produced annually.

From the data presented above, it is seen that lignocellulosic sources are released in enormous quantities and can be used as a substrate for fermentative production of various value-added chemicals after individual sugars liberating.

## 4 Pretreatment Methods

**FIGURE 5:**
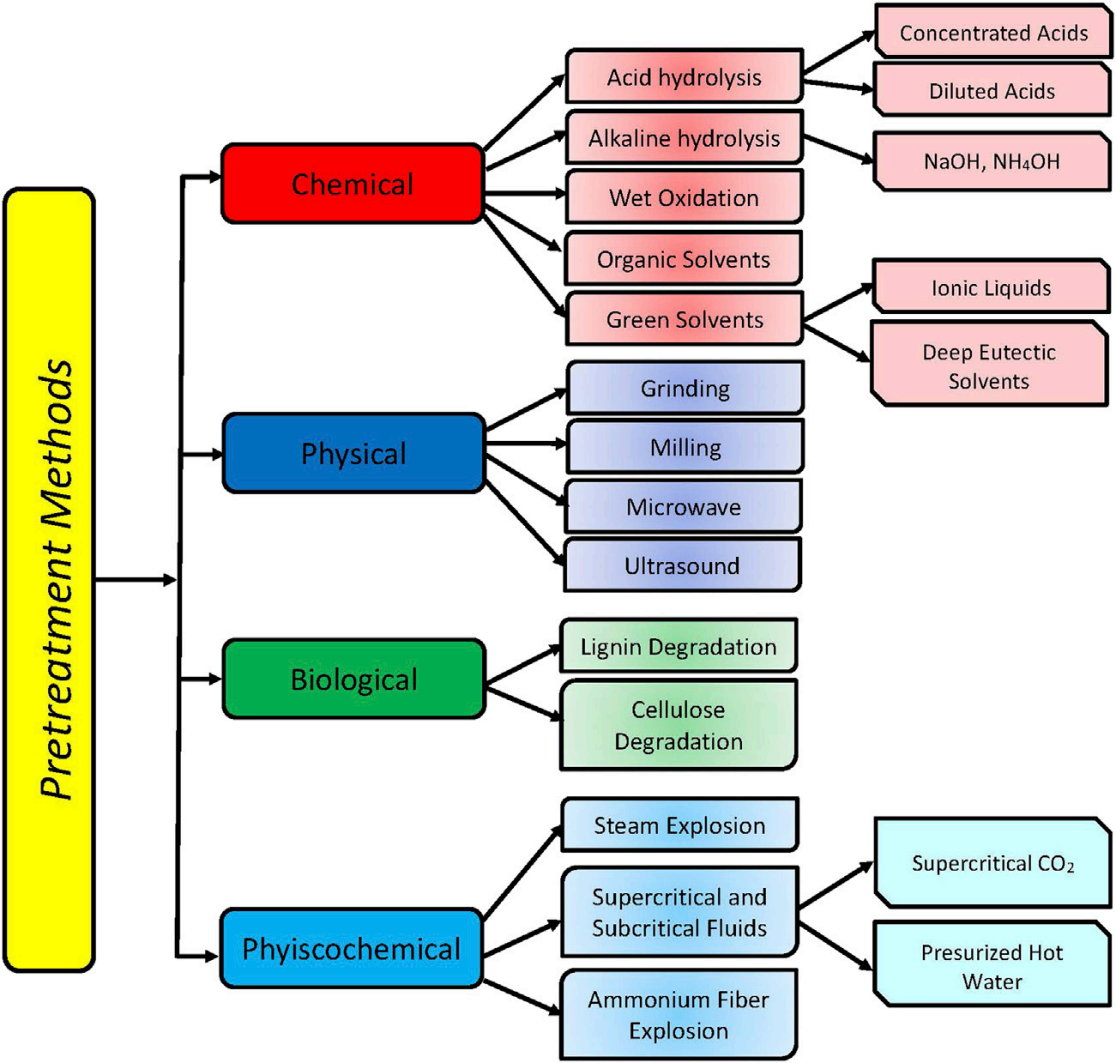
Classification of biomass pretreatment methods.

The specific structure of the lignocellulose restricts the access of enzymes to cellulose and hemicellulose fibers and hinders effective hydrolysis. In view to converting lignocellulose to fermentable sugars, the lignocellulosic biomass must be subjected to some pretreatment. The purpose of the pretreatment is to break down the structure of lignocellulose, destruct hydrogen bonds between fibers, remove at least partially the lignin, decrease the crystallinity of cellulose, and degree of polymerization, and thus increase the area accessible for the enzymes’ action, as well as efficiently hydrolyze cellulose and hemicellulose. Different methods have been applied for the pretreatment of lignocellulosic biomass (Norrrahim et al., 2021). In general, they can be classified into four main groups—physical, chemical, physicochemical, and biological methods (Figure 5). When the main goal of lignocellulosic biomass processing is obtaining maximum fermentable sugars as a substrate for fermentative production of value-added products, pretreatment is an inevitable step. Usually, each method results in different effects (delignification, hemicellulose dissolution, etc.). The most important is that there is no universal method for lignocellulosic biomass pretreatment. This is due to the different compositions of lignocellulosic biomass (ratio of main components and degree of crystallinity of cellulose) from different sources (soft- and hard-wood, agricultural, or industrial wastes). The compositional differences influence the efficacy of pretreatment. The severity of treatment (pressure, temperature, and acid or alkali concentration) also lead to differences in the composition of obtained hydrolysates as well as in the concentration of inhibitors of enzyme hydrolysis and fermentation. Recently Zhao et al. (2022) summarized the current state and the latest advances of lignocellulose pretreatment technologies and described several new strategies for overcoming pretreatment bottlenecks for the realization of highly efficient lignocellulose bioconversion.

In view to choosing an “optimal” pretreatment method, one needs broad knowledge about the chemical composition of the feedstock, but also the final aim of pretreatment. For example, in case the fermentation step is done by an industrial strain non-converting pentoses—lignin, and hemicellulose should be separated from biomass before cellulose hydrolysis. In the case of using a strain capable to convert pentoses or co-fermentation removal of lignin should be enough. Usually, a combination of two or more methods is used before the enzymatic hydrolysis of lignocellulosic biomass.

### 4.1 Physical Methods

The main purpose of these methods is to reduce the particle size of biomass, the crystallinity of cellulose, and the degree of polymerization of cellulose and hemicellulose and thus increasing surface area accessible for enzymes and biomass biodegradability. Physical methods do not use any chemicals and include different types of chipping, milling, and grinding, as well as gamma- or microwave irradiation, and ultrasound treatment. The main disadvantage of physical methods is high energy requirements, especially on a large scale.

### 4.2 Chemical Methods

This group includes several methods, based on reactions between lignocellulose biomass and various chemicals in the water phase. This is the most applied group of methods and its main goal is to reduce the amount of lignin and hemicellulose content in the biomass.

#### 4.2.1 Acid and Alkaline Hydrolysis

These methods are relatively cheap and easy to perform. Acid hydrolysis of biomass can be done using diluted (0.5–5%) or concentrated (10–60%) acids. Sulphuric and hydrochloric acids are most employed. Smits et al. (2019) developed a fast method for screening of optimal acid-pretreatment conditions in the conversion of wood lignocellulose to sugar. Luo et al. (2021) developed an artificial neural network model to simultaneously predict the derived feature of phenolic compounds content and glucose yield in lignocellulosic biomass hydrolysate from dilute inorganic acid pretreatment and enzymatic hydrolysis. Also, there are attempts to use some organic acids to avoid problems with the corrosive action of the acids. While hydrolysis with concentrated acids can be realized at low temperature and normal pressure, dilute acid hydrolysis require elevated temperatures and pressure.

Hydroxides like NaOH, KOH, NH_4_OH, Ca(OH)_2_. Hydroxides are less corrosive in comparison to acids and can be applied at moderate temperatures and ambient pressure when the alkali concentration is about 10–20%. At low concentrations higher temperatures and elevated pressure are necessary. These methods are considered better in deligninfication compared to acid hydrolysis methods, whereas cellulose remains mostly intact, and hemicellulose is affected depending on the severity of treatment.

Another successful method for lignin separation is so-called solvolysis. This technique uses some low boiling alcohols (methanol, ethanol) or high boiling alcohols, like glycerol, ethylene glycol, and butanol. Some ketones—acetone, methyl isobutyl ketone, and organic acids (formic, acetic, oxalic) are also utilized. Methods applying low boiling chemicals require high temperatures (150–250°C) and pressure. After solvolysis pretreatment, the lignocellulosic biomass separates into three fractions—precipitated lignin, solid cellulosic pulp, and liquid phase, containing hemicellulosic sugars (Goh et al., 2011). Sidiras et al. (2022) reviewed organosolv pretreatment methods and the optimization of feedstock delignification, sugars production, enzymatic digestibility of the cellulose fraction, and quality of lignin.

#### 4.2.2 Oxidative Pretreatment

Oxidative pretreatment methods involve biomass processing with air, pure oxygen, hydrogen peroxide, or ozone in the water phase. In the case of ozone, the process can be conducted at ambient conditions, while other oxidizers demand high temperatures (150–250°C) and pressure—10–20 bar for short periods—10–15 min. During pretreatment, the lignin is converted to carboxylic acids, carbon dioxide, and water. The presents of acids in the solution facilitate the solubilization of the hemicellulose and increase the cellulose content.

#### 4.2.3 Ionic Liquids and Deep Eutectic Solvents

In the last 20 years, the application of ionic liquids (IL) and deep eutectic solvents (DES) in biomass pretreatment gains more and more the attention of researchers. Ionic liquids are composed of a large organic cation and a small anion. They are liquid at room temperature with low vapor pressure, which facilitates their almost full recovery and thus somehow overcomes the main drawback of using IL—their high cost. Deep eutectic solvents are easily prepared by heating (60–80°C, for 4–8 h) a hydrogen bond donor (urea, acetamide, glycerol, some acids—lactic, oxalic, and sugars—glucose, maltose) and a hydrogen bond acceptor (different quaternary ammonium salts—choline chloride, tetramethylammonium chloride, etc.). The compounds forming DES are linked through hydrogen bonds and the formed eutectic mixture has a lower melting temperature than the initial components. The main advantages of DES are their ease of synthesis, biodegradability, and low cost. It is accepted that IL and DES are more effective in the separation of lignin and hemicellulose than acid and alkali treatment. Xu et al. (2021) analyzed the influence of different hydrogen bond donors on the lignocellulosic biomass pretreatment with choline chloride-based DES. The authors defined the most important among 42 key process factors—HBD hydrophilic ability, HBD polarity, HBD acidity, HBD ability to form hydrogen bonds, the molar ratio of HBD to choline chloride, and pretreatment severity (Xu et al., 2021). Yadav et al. (2020) have screened some lactic acid bacteria for their stability in ionic liquids. Recent reviews published by Usmani et al. (2020) and Halder et al. (2019) describe recent developments in using techniques applying ionic liquids in biomass pretreatment—breakdown mechanism, process parameters, the impact of cation and anion groups, and the advantages of IL on the further processing of the fractionated biomass.

### 4.3 Physicochemical Methods

These methods combine physical forces with the action of chemicals to modify the lignocellulosic biomass structure. Steam explosion, ammonia fiber expansion (AFEX), and supercritical fluids treatment take part in this group. The treatment can be done with pure water or water steam at elevated pressure and temperatures (hydrothermal treatment) or in the presence of some chemicals acting as a catalyst. Depending on temperature and pressure (bellow or above the critical point of the solvent) methods can be divided into two groups—subcritical and supercritical. As a result of physicochemical pretreatment dissolution of hemicellulose, changes in lignin structure and decreasing degree of polymerization and cellulose crystallinity and are observed. Duque et al. (2016) described the fundamentals of steam explosion methods, their advantages and disadvantage, and technological advancements made in the last years.

#### 4.3.1 Steam Explosion

In this method the biomass is subject to high temperature (150–250°C) for a short time (from certain seconds to a few minutes) in the presence of saturated steam, followed by a quick release of the pressure. Sometimes a two-step method is applied—in the first step, the biomass is heated to 180°C in view to solubilize and remove hemicellulose, and then the temperature is elevated to 240°C to modify cellulose and lignin. The method is environmentally friendly with advantages like low energy consumption and low cost. The drawback is the formation of some fermentation inhibitors.

#### 4.3.2 Pressurized Hot Water

Similarly to steam explosion the biomass is heated to 150–250°C at pressure 3–5 MPa, but without quick decompression, which permits to realize the process in a flow-through reactor. The method is cost-effective, does not use toxic chemicals, and is possible to control the release of inhibitors by controlling pH values between 4 and 7.

#### 4.3.3 Ammonia Fiber Expansion

The method uses liquid or gaseous ammonia (usually at 1:1 ratio) under high pressure (15–30 bar) and moderate temperatures (60–100°C) for 5–10 min. The moderate temperatures, as well as the possibility of recovering and reuse of the ammonia, reduce the cost of the process. The method is not very effective in treating biomass with high lignin content (higher the 20%) and also does not fully dissolute the hemicellulose. Recently, a new method called extractive ammonia is investigated. It is characterized by a higher ratio of ammonia to biomass (up to 6:1). The method is very effective in lignin removal (about 45% in corn stover) leaving almost all carbohydrates (more than 90%) in the solid phase (Avci et al., 2019).

#### 4.3.4 Supercritical Fluid Pretreatment

Supercritical fluids are characterized by their unique properties—they have a density like a liquid and viscosity and diffusivity like gases. Carbon dioxide is the most widely used chemical in supercritical conditions. While the biomass pretreatment with sec carbon dioxide is not enough effective, the presence of moisture ameliorates changes in the biomass structure significantly. In the presence of moisture, CO_2_ forms carbonic acid which augments hemicellulose hydrolysis. Supercritical CO_2_ can be used also with co-solvents (methanol or ethanol) and thus enhance digestibility. The main advantages of this method are low cost, easy recovery of CO_2_, no release of toxic compounds, non-inflammability of CO_2_, and reduced environmental impact. Supercritical water is also used for biomass pretreatment. In contrast to supercritical CO_2_, in the case of water, much higher temperatures and pressures are applied. Short reaction times (1–2 s) result in high sugars yield, whereas long reaction times decrease the yield.

### 4.4 Biological Methods

These methods involve the use of enzymes or enzymatic systems of whole microorganisms (mainly fungi and bacteria. Depending on their goal, the methods can be divided into two groups—delignification and saccharification methods. Fungi (white-, brown and soft-rot) possess in their enzymatic system enzymes like lignin peroxidases, manganese peroxidases, laccases, glyoxal, and alcohol oxidases and are capable to degrade lignin. Some fungi degrade selectively lignin (mainly white-rot), while others attack also cellulose and hemicellulose (Rouches et al., 2016). Except for fungi, there are many bacterial strains (*Bacillus* sp., *Streptomyces* sp., *Clostridium* sp., *Thermomonospora* sp., *Cellulomonas* sp., etc.), producing enzymes capable to degrade various lignocellulosic biomasses (Sharma et al., 2019). Besides using an individual strain in biomass pretreatment, very often consortia of different microorganisms are applied. Working with consortia can provide process robustness, higher stability, and productivity of the treatment. Recently, the application of lignin-degrading enzymes instead of using whole organisms gains considerable interest among researchers. The most utilized enzymes belong to cellulases, ligninases, and xylanases. Different enzymes like laccase, versatile peroxidase, manganese peroxidase, and lignin peroxidase are effective in lignin degradation (Kumar and Chandra 2020). Cellulases are multienzyme complexes comprising mainly three components: endo- β 1–4—glucanase, exo- β 1–4—glucanase, and β-glucosidase. Endo- β 1–4—glucanase hydrolyzes internal links in cellulose chains to short-chain cellodextrins. Exo- β 1–4—glucanase cleaves cellobiose from the non-reducing end of the chains, and β-glycosidase hydrolyzes cellobiose units to glucose units (Shuddhodana et al., 2016). Regarding the heteropolysaccharide structure of hemicellulose, different enzymes are needed to hydrolyze it to monomeric sugars. Endo-xylanases and endo-mannanases randomly attack the inner bonds in xylan and mannan, while β-xylosidase and β-mannosidase hydrolyze oligosaccharides to xylose and mannose. For hydrolysis of the lateral groups (arabinose, acetyl, galactose, and glucose) linked to the main polysaccharide chain supplementary enzymes are necessary—α-glucuronidase, acetyl xylan esterase, acetyl mannan esterase, ferulic acid esterase, etc. (Laca et al., 2019).

Biological methods of lignocellulosic materials degradation have advantages, such as low energy cost, environmental friendliness, low capital investment cost, no reagent dependence, and no production of fermentation inhibitors. Still, long reaction time and low reaction rate, and the relatively high price of the enzymes are the main limitations, preventing the successful implementation of the biological methods in the industry.

Recent trends in lignocellulosic biomass pretreatment methods, their advantages, and disadvantages and the role of various key factors that affect the separation of lignocellulose constituents are discussed in Baruah et al. (2018) and Mankar et al. (2021). It is worth mentioning that the use of only one pretreatment method is not effective and usually a combination of two or more methods are applied. It seems that the use of a mechanical method for reducing the size of the biomass particles and enzyme hydrolysis of hemicellulose and cellulose to fermentable sugars is inevitable.

The most important is that there is no universal method (or combination of methods) for lignocellulosic biomass pretreatment. This is due to the different compositions of lignocellulosic biomass (ratio of main components and degree of crystallinity of cellulose) from different sources (soft and hardwood, agricultural or industrial wastes). The compositional differences influence the efficacy of pretreatment. The severity of treatment (pressure, temperature, and acid or alkali concentration) also lead to differences in the composition of obtained hydrolysates as well as in the concentration of inhibitors of enzyme hydrolysis and fermentation. In view to choosing an “optimal” pretreatment method, one needs broad knowledge about the chemical composition of the feedstock, but also the final aim of pretreatment. For example, in case the fermentation step is done by a strain not converting pentoses—lignin and hemicellulose should be separated from biomass before enzyme hydrolysis. In case of using a strain capable to convert pentoses or co-fermentation removal of lignin should be enough. In general, an “ideal” lignocellulose pretreatment method should provide 1) full degradation and removal of the lignin presented; 2) utterly separation of cellulose and hemicellulose; 3) possibility of complete liberation of fermentative mono sugars; 4) minimal or no release of fermentation inhibitory compounds.

Recently Shen and Sun described three strategies for lignocellulosic biomass pretreatment, depending on the final goal, namely cellulose-first, hemicellulose-first, and lignin-first strategies together with most appropriated methods. These strategies possess unique advantages, permitting a component of lignocellulose to be selectively fractioned for value-added application (Shen and Sun 2021).

Table 2 summarizes the advantages and disadvantages of different pretreatment methods and Table 3—the methods used in lignocellulosic biomass pretreatment for lactic acid production.

**TABLE 2:** Advantages and disadvantages of different pretreatment methods.

Pretreatment methods		Process	Advantages	Disadvantages
	Physical	Milling	No chemicals use; no inhibitory compounds or byproducts formation; Reduce biomass size, degree of polymerization; and crystallinity of cellulose; short operation time. Increase surface area for enzymatic hydrolysis	Low sugar yield; High-energy requirements; No lignin degradation
		Grinding		
		Chipping		
		Gamma-or microwave irradiation		Special equipment design; High costs in large scale
		Ultrasound treatment		
	Chemical	Acid	High reaction rate and relatively short time; Remove hemicellulose and partly lignin; Increase surface area for enzymatic hydrolysis; High yield of sugars after enzymatic hydrolysis	High cost; Inhibitors formation, Need of anticorrosive equipment and neutralization; Sugar loses
		Alkaline	Moderate reaction conditions; Less corrosive than acids; Delignification; Hemicellulose is affected depending on severity of treatment; Decrease degree of polymerization; and crystallinity of cellulose	High costs; Salts formation; Relatively long reaction time
		Oxidative	Lignin degradation; Hemicellulose solubilization; Low inhibitors formation; Possibility of working at ambient conditions	Elevated cost when using ozone or hydrogen peroxide; Some oxidants are toxic or corrosive
		Organosolv	Lignin and hemicellulose solubilization; Lignin recovery; High sugars yield; Increase surface area for enzymatic hydrolysis	Expensive; Energy consuming; Need for solvents regeneration; Inhibitors generation; Explosion and fire dangers
		Ionic liquids (IL) and deep eutectic solvents (DES)	Mild conditions; Very good lignin separation; High biomass loading; High cellulose solubilization; DES are biodegradable and biocompatible; No toxic product formation	Need for recovery and recycle; Possible enzyme inhibition; High IL cost
	Physicochemical	Steam explosion	Environmentally friendly; Low energy consumption; Cost effective; Short process time; High sugars yield in two step method	High pressure and temperatures; Special equipment; Toxic and inhibitory compounds formation; Partial hemicellulose degradation; Less effective at high lignin content
		Pressurized hot water	Cost-effective; No use of toxic chemicals; Possibility to control inhibitors release by controlling pH; Low or no inhibitors release	Long residence time; High temperatures and pressure; Special equipment; Less effective at lignin removal
		Supercritical CO_2_	Non-toxic chemicals used; Increases accessible surface area for enzyme hydrolysis, High solid load; No inhibitors formation	High pressure; High equipment cost; Does not modify lignin; Less effective at high lignin content
		AFEX	Short process time; Possibility of recovering and reuse of the ammonia; Efficient removal of lignin; Low formation of inhibitors	Less efficient for biomass with high lignin content; partial dissolution of hemicellulose; Large amount of used ammonia
	Biological	Microbial	Low energy consumption; Mild reaction conditions; No chemicals use; No formation of inhibitors; Efficient lignin and hemicellulose degradation; Environmentally friendly	Slow reaction rate; Long process time; High cost of enzymes; Necessity of strict and sterile conditions
		Enzymatic		

**TABLE 3:** Pretreatment methods and conditions of different lignocellulosic biomasses.

Pretreatment method	Lignocellulosic biomass	Conditions	Reference
Ionic liquids pretreatment			
[EMIM][Ac] 1-Ethyl-3-ethylimidazolium-acetate	Cottonseed cake	Biomass to IL ratio of 1:2 (w/w); at 120°C for 2 h	Grewal and Khare (2018)
	Wheat straw		
	Sugarcane bagasse		
[EMIM][OAc] 1-Ethyl-3-methyl-imidazolium-acetate	Rice straw	2:1, 1:1, 1:2, 1:3 Biomass to IL ratios at 120°C for 2 h	Yadav et al. (2021)
([Emim][Ac] 1-Ethyl-3-methylimidazolium Acetate	Wheat straw	Biomass to IL ratios of 1:1, 1:3, and 1:5, 120°C for 120 min	Marin-Batista et al. (2021)
	Barley straw		
	Grape stem		
Pyridiniumhydrogensulphate	Wheat straw	Lignin extraction—60°C, 2 h, with 5% biomass loading	Asim et al. (2021)
		Reducing sugars—100°C, 2 h with 5% biomass loading	
Deep eutectic solvents			
Triethylbenzyl ammonium chloride/lactic acid	Wheat straw	Solid:liquid ratio—1:15, 100°C for 10 h	Liu et al. (2019)
Ethylammonium chloride/ethylene glycol—enzymatic	Oil palm trunk	100°C, 48 h	Zulkefli et al. (2017)
		Celluclast 1.5 L—53 FPU/ml Novozyme 188—122 CBU/ml, 50°C, 24, pH 4.8	
Choline chloride/glycerol with salts—enzymatic hydrolysis	*Pennisetum* biomass	Five times at 120°C for 6 h	Wang et al. (2020)
		Cellic CTec2, pH 4.8, 15 FPU/ml, 72 h	
Choline chloride/glycerol	Coffee silverscin	150°C, 3 h, biomass:solvent ratio 1:32	Procentese and Rehmann (2018)
2-aminoethanol, 2-(methylamino)ethanol, 2-(ethylamino)ethanol, diethylamine, triethylamine and butan-1-amine with sulphuric and acetic acid	Sugarcane bagasse	IL:H_2_O ratio 5:1, 160°C, 3 h	Pin et al. (2019)
Sequential pretreatment choline chloride:urea (1:2 ratio) and divalent inorganic salt (CuCl_2_)	Oil palm fronds	Solid:liquid ratio 1:10	Loow et al. (2018)
		120°C, 4 h	
		CuCl_2_ 120°C, 30 min	
Chemical pretreatment			
Sulfuric acid	Exhausted sugar beet pulp	Solid/liquid ratio 1:20, 1%, 120°C, 20 min	Marzo et al. (2021)
Sulfuric acid/enzymatic hydrolysis	Wheat straw	0.5% H_2_SO_4_ at 180°C for 4 min	Cubas-Cano et al. (2019)
		Cellic-HTec2 and Megazyme	
		40°C, pH 5.5 for 72 h	
Sulfuric acid or enzymatic hydrolysis	Wheat straw	4% H_2_SO_4_ at 120°C for 30 min	Cubas-Cano et al. (2019)
		40°C, pH 5.5 for 24 h	
HCl treatment/cellulose hydrolysis	Corn straw	6% HCl at 90°C for 1°h, cellulase complex-solid-liquid ratio 1:20 at 50°C for 48 h	Si et al. (2020)
Sulfuric acid/enzymatic hydrolysis	Corn stover	2% sulfuric acid at 130°C, 8 min	Utrilla et al. (2016)
Sulfuric acid/enzymatic hydrolysis	Distillers’ dried grains with solubles	3 M H_2_SO_4_ at 110°C for 24 h	Krull et al. (2020)
		1% enzyme solution, 60°C, 48 h	
Sulfuric acid hydrolysis	Sugarcane bagasse	2% sulfuric acid at 121°C; 1.5 h; 2:1 liquid:solid ratio	Utrilla et al. (2016)
Sulfuric acid hydrolysis	Sugarcane bagasse	2% sulfuric acid at 122°C for 24 min, solid:liquid ratio of 1:8	González-Leos et al. (2020)
Sulfuric acid hydrolysis	Rice straw	1% sulfuric acid at 180°C for 1 min, 40% solid content	Tu et al. (2019)
Sulfuric acid hydrolysis	Microalgae *Chlorella vulgaris* ESP-31	4% H_2_SO_4_, 120°C, 20 min, continuous fermentation, PVA immobilized cells	Nandini et al. (2020)
Sulfuric acid hydrolysis	Green seaweed *Enteromorpha prolifera*	0.5 M H_2_SO_4_ at 120°C for 2 h	Hwang et al. (2012)
Sulfuric acid hydrolysis	Spent coffee grounds	1% H_2_SO_4_ at 121°C for 30 min	Kim et al. (2019)
Sulfuric acid hydrolysis	Corn stover	7.2% H_2_SO_4_ at 175°C for 5 min solid/liquid ratio -2:1	Wei et al. (2018)
Sulfuric acid hydrolysis	Corn stover	2% H_2_SO_4_ at 160°C for 60 min 10% solid loading	Jiang et al. (2016)
Oxalic acid hydrolysis	Corn cob	5% oxalic acid, 25°C for 30 min	Alhafiz et al. (2020)
		1:10 solid: liquid ratio	
Acid-catalyzed steam-exploded hydrolysis	Corn stover	1.29% H_2_SO_4_ at 175°C for 5 min	Jiang et al. (2016)
Liquid hot water hydrolysate	Corn stover	180°C for 40 min, 1:10 solid:liquid ratio	Jiang et al. (2016)
Sulfite hydrolysis	Corn stover	4% (Mg(HSO_3_)_2_ at 160°C for 60 min, 1:6 solid:liquid ratio	Jiang et al. (2016)
Sulfuric acid/enzymatic hydrolysis	Spent coffee grounds	27 g/L H_2_SO_4_, 121°C, 40 min	Hudeckova et al., (2018)
		4 vol% of Celluclast 1.5 L, 0.4% β-glucosidase, 0.4% Viscozyme L 50°C, 48 h	
Sulfuric acid/enzymatic hydrolysis	Wheat straw	1.5% sulfuric acid for 40 min at 160°C, solid–liquid ratio 1:10	Ouyang et al. (2020)
		Cellulase Cellic Ctec2, 5 FPU/g cellulose, 150°C, pH 5.5, 150 rpm for 72 h	
Hydrochloric acid hydrolysis	Sugarcane bagasse	(v/v) of HCl, at 140°C, for 15 min, 10% solid load of	Oliveira et al. (2019)
HCl/cellulase hydrolysis	Marine algae *Gracilaria* sp., *Sargassum siliquosum*, *Ulva lactuca*	0.4 N HCl, 30 min at 121°C; 7.6 U/mL cellulase at 37°C for 48 h	Lin et al. (2020)
Acid pressurized hydrolysis	Rice husks	175°C (58.8 bar), 46 min, 0.8% HCl or 2.2% H_2_SO_4_	Montipo et al. (2016)
	Agave bagasse		
Phosphoric acid/enzymatic hydrolysis	Rye straw	75–80% phosphoric acid, 50–75°C, ratio 1:3 15–45 min	Schroedter et al. (2021)
	Digestate of energy corn silage	Cellic^®^ CTec 2,0.4 ml/g, 11.2% total solids, 50°C, 24 h	
NaOH/cellulase hydrolysis	Corn Stover	5% NaOH, 75°C for 3 h, 20% solid loading	Hu et al. (2016)
		Cellic CTec2	
NaOH/cellulase hydrolysis	Sweet sorghum bagasse	118°C for 80 min 2% (w/v) NaOH at a 10% (w/v) loading	Wang et al. (2016)
		Cellulase—25 FPU/g	
NaOH/cellulase hydrolysis	Sugarcane bagasse	0.5 M NaOH at 80°C; 120 min	Nalawade et al. (2020)
		Cellulase—50°C, 24 h	
NaOH/cellulase hydrolysis	Corn stover	5% NaOH at 75C for 3 h at a 20% solid loading	Zhang et al., (2020)
		Cellic CTec2	
NaOH/cellulase hydrolysis	Corn stover	2% NaOH, solid:liquid ratio 10% at 118°C for 1 h	Chen et al. (2020)
		Cellulase 20 U/g solid in SSF	
NaOH/cellulase hydrolysis	DDGS	5% NaOH at 121°C for 15 min at 10% DDGS loading	Zaini et al. (2019)
		Cellulase-Accellerase^®^ 1,500 ratio enzyme:cellulose 3:1 at 50°C	
NaOH/cellulase hydrolysis	*Sophora flavescens* root	1.5% NaOH, 120°C, 2 h, solid/liquid ratio 1:10.25 FPU/g, 50°C 72 h	Ma et al. (2020)
Alkaline peroxide/cellulase hydrolysis	Corn stover	NaOH solution, and 33.3 g H_2_O_2_ (30%), solid:liquid ratio 1:20 for 1 day	Akao et al. (2016)
		Cellulase—Meiselase 60 U/g during SSF	
Alkaline/cellulase hydrolysis	Date palm wastes	2N NaOH, 50°C, 48 h	Alrumman (2016)
		4% substrate, 30 FPU/g, pH 5.0	
Alkaline/peroxide	Corn stover	3% NH_3_OH for 2 days, 5% H_2_O_2_ for 7 days at room temperature	Liu et al., (2020)
Alkaline/Hydrogen Peroxide	Exhausted sugar beet pulp	Solid/liquid ratio 1:20, 1% hydrogen peroxide, pH 11.5, 30°C, 24 h	Marzo et al. (2021)
KOH/enzyme hydrolysis	Spend coffee grounds	35 g/L of KOH, 121°C, 60 min	Koo et al. (2019)
		Viscozyme L -2%, 55°C, 7 days	
KOH/enzyme hydrolysis	Spend coffee grounds	3% KOH, 75°C, 2.8 h cellulase, cellobiase, and mannanase—3% solid loading, 50°C, 96 h	Lee et al. (2021)
Ammonia/enzyme hydrolysis	Corn stover	15% ammonia, 0.3 wt% polyDADMAC, solid/liquid ratio 1:9, 160°C, 1 h	Shi et al. (2021)
		5 FPU/g- Cellic^®^ C-Tec2, 50°C, and 200 rpm, 120 h	
Acid, alkali, and enzyme hydrolysis	Sugarcane bagasse	solid:liquid ratio of 1:2.8, 1% sulfuric acid at 121°C, 27 min	Wischral et al. (2019)
		4% sodium hydroxide at 121°C, 30 min, solid:liquid ratio 1:20	
		Enzymatic hydrolysis at 50°C, pH 5.0, 24 h with a commercial enzyme cocktail	
Hydrothermal pretreatment with magnetic carbon-based solid acid followed by enzyme hydrolysis	Sugarcane bagasse	sugarcane bagasse, catalyst and water in ratio s 1:1:25 (g:g:mL), 170°C for 10 min	Lu et al. (2021)
		Cellulase—at 50°C for 72 h	
Wet explosion and enzyme hydrolysis	Corn stover	190°C, 30 min, oxygen loading- 7.5%	Ahring et al. (2016)
		Cellic^®^ CTec2-50°C, pH 5.0, 4 days	
Hydrothermal pretreatment with ethylenediamine	Rice straw	80–200°C, solid; liquid ratio 1:10 for 1 h	Chen et al. (2019)
Ethanolysis	Corn stover	Ethanol:H_2_O (1:1) co-solvent 0.050 M oxalic acid at 140°C for 1 h	He et al. (2016)
Acid-organosolv	Sugar palm trunk	0.2 M H_2_SO_4_ solid:liquid ratio 1:5,120°C 40 min	Erliana et al. (2020)
Biological pretratment			
Enzyme hydrolysis	Microalga *Arthrospira platensis*		Werlang et al. (2020)
α-amylase		2 h at 90°C	
glucoamylase		2 h at 60°C	
cellulase		40 h at 50°C	
Enzyme hydrolysis α-amylase, glucoamylase, cellulase	Cassava bagasse	60 min at 105°C	Chen et al. (2020)
		Glucoamylase and cellulose are added during SSF	
Enzyme hydrolysis α-amylase, glucoamylase, cellulase	Brewer’s spent grain	Termamyl SC^®^ (α-amylase),1 h at 90°C), SAN Super 240 L^®^ (amyloglucosidase, α-amylases), 1 h at 55°C	Pejin et al. (2017)
		Celluclast 1.5 L^®^ (cellulase) 10 h at 45°C	
Enzyme hydrolysis	Corn stover	Crude lignocellulolytic enzyme system	Ma et al. (2016)
		50°C, pH 5.0, 72 h	
Enzyme hydrolysis	Rice straw	Cellic CTec2 (20 units/1 g), 7.5%, pH 5), at 50°C, 24 h	Verma and Subudhi (2021)
Enzyme hydrolysis—Celluclast 1.5; Novozym188; Pectinex Ultra SP-L	Orange peel	pH 5.2; 50°C, for 26 h	de la Torre et al. (2017)
Enzyme hydrolysis—Celluclast 1.5; Novozym188; Pectinex Ultra SP-L	Orange peel waste	pH 5.2; 50°C, for 26 h	Bustamante et al. (2020)
Enzyme hydrolysis—Cellic^®^ CTec2	Pulp mill residue	Solid:liquid ratio 1:4, 25 FPU/g, pH 5.0; 50°C 26 h	Moraes et al. (2016)
Enzyme hydrolysis—Cellic^®^ CTec2	Date pulp waste	150 g/L total solids, pH 5.0–5.5, 50°C, 72	Ahmad et al. (2021)
Solid state fermentation with *Aspergillus awamori* 2B.361 U2/1	Exhausted sugar beet pulp	Moisture content 70%, 30°C, 8 days	Marzo et al. (2021)
Enzyme hydrolysis Viscozyme^®^ and Ultraflo®Max	Sugar beet pulp	12% pulp, Viscozyme^®^: Ultraflo®Max—1:1, 37°C, 16 h	Berlowska et al. (2016)
Enzyme hydrolysis commercial and fabricated cellulases (1:1)	Paper sludge	2.4 FPU/g, pH 6.0, 40°C, 144 h	Dhandapani et al. (2021)
Physical pretreatment			
Microwave-assisted autohydrolysis/cellulase (Ctec2) hydrolysis	Macroalgae *Eucheuma denticulatum*	120°C and 50 min/pH 4.8 at 50°C for 72 h	Chai et al. (2021)
Mechanical screw press	Sweet sorghum	Chopping <20 mm and pressure of 5 bar	Olszewska-Widdrat et al. (2019)
Physicochemical pretreatment			
Steam explosion	Corn stover	Solid/liquid ratio 2:1, 1.6 MPa, 201°C, 5 min	Han et al. (2021)
Steam explosion	Beech wood chips	230°C, 15 min	Shahab et al. (2018)
Organosolv	Wheat straw	80°C, 70% formic and acetic acids 1:1, 30 min	Tsegaye et al. (2021)
Supercritical CO_2_	Cyanobacterium *Arthrospira platensis*	450 bar, 40°C, CO_2_ flow 4 g/min	Esquivel-Hernández et al. (2021)

## 5 Detoxification

As was mentioned above, the majority of pretreatment methods generated some products that can hinder subsequent hydrolysis and fermentation. The inhibitory compounds are a result of the degradation of lignin and sugars from cellulose and hemicellulose and represent different carboxylic acids and also furanic and phenolic compounds. Hexoses from cellulose and hemicellulose can be transformed during pretreatment to 5-hydroxymethyl furfural (HMF), which further dehydration leads to levulinic and formic acid formation. Various inhibitory products like furan aldehydes, sugar acids, and aliphatic acids are produced upon hemicellulose pretreatment. Acetic, formic, acrylic, and levulinic acids are detected in hemicellulose hydrolysates, but furfural is the most strong and abundant inhibitor. Various phenolic compounds are liberated during lignin pretreatment. The concentration and type of these compounds depend on biomass source and H/G/S group ratio in the lignin structure. The most important lignin-degradation products are *p*-coumaric acid, ferulic acid, 4-hydroxybenzoic acid, vanillic acid, syringic acid, 4-hydroxybenzaldehyde, syringaldehyde, vanillin, etc. In a recent review paper, Kumar et al. summarized various inhibitory compounds produced during lignocellulosic biomass pretreatment and different methods for hydrolysates detoxification and inhibitors removal (Kumar et al., 2020). Vanmarcke et al. (2021) also have described the inhibitor composition of different lignocellulose hydrolysates resulting from different pretreatment methods and investigated individual inhibitor effects in the case of ethanol fermentation.

Van der Pol et al. (2016) developed a small-scale rapid screening method to identify inhibitory effects of single and combined by-products from acid and alkaline hydrolysates on the growth of lactic acid-producing microorganisms. Five lactic acid-producing strains were tested—*Lactobacillus casei*, *Lactobacillus delbrueckii*, *Lactococcus lactis*, *Bacillus coagulans*, and *Bacillus smithii*. While in the presence of alkaline treatment by-products, *L. caseis* was least affected (depending on the inhibitor’s concentration), in the case of acid treatment—only growth of *L. casei* and *L. lactis* was not fully inhibited. The synergy between formic acid, acetic acid, and coumaric acid was found as a key inhibitory parameter in alkaline pretreated lignocellulose, while in the case of acid pretreated lignocellulose, furfural was the major inhibitor (van der Pol et al. (2016)). Cola et al. (2020) investigated the influence of an inhibitory cocktail (acetic acid, HMF, furfural, and *p*-coumaric acid) on the growth of four *Saccharomyces cerevisiae* and four *Lactobacillus paracasei* strains. Whilst all yeast strains were unable to grow in the presence of inhibitory compounds from sugarcane-based lignocellulosic hydrolysates, bacteria preserved an average of roughly 50% of their growth rates.

In view to reducing the content of inhibitory compounds, different approaches have been used. Various physicochemical methods like adsorption, ion exchange, extraction, membrane separation, overliming, etc. are applied. Other strategies involve biological treatment of the hydrolysates, gene manipulation of the microorganisms to improve their tolerance in regards to inhibitors, or plant improvement to decrease lignin content. Biological detoxification can be mediated by microorganisms or enzymes like laccase (Bhatia et al., 2020). Tramontina et al. (2020) reviewed enzyme-mediated detoxification methods for the removal of inhibitory compounds from lignocellulosic hydrolysates and described some novel strategies using classical enzymes such as laccases and peroxidases, as well as more advanced strategies using prooxidant, antioxidant, and detoxification enzymes, i.e., superoxide dismutases.

Ludwig et al. (2013) investigated detoxification of organosolv pretreated wheat straw by using free and immobilized laccase. Laccase was immobilized on Sepabeads (modified methacrylic polymer) ion-exchange resin. Free laccase was capable to remove up to 82% of phenolic compounds, while immobilized laccase could remove also HMF and acids not only by enzyme action but by the polymerization and *in-situ* product removal. Phenolic compounds precipitated onto the carrier surface and could be easily removed. In all cases, enzyme treatment improved the fermentability of pretreated wheat straw hydrolysate (Ludwig et al., 2013).

Liu et al. (2019) have isolated three strains (*Enterococcus faecalis* B101, *Acinetobacter calcoaceticus* C1, and *Pseudomonas aeruginosa* CS) capable to utilize some phenolics from ammonia pretreated corn stover (vanillin, 4- hydroxybenzaldehyde, or syringaldehyde) as a sole carbon source. Lactic acid production from 50 g/L ammonia pretreated corn stover was increased nearly twofold [from 16.98 g/L to 31.35 g/L LA (0.63 g/g corn stover)] by inoculating phenolic degrading bacteria mentioned above and lactic acid bacteria *Lactobacillus pentosus* FL0421 (Liu et al., 2019). In another paper, Liu et al. (2020) cultivated T*richoderma viride* R16 on alkaline/peroxide pretreated corncob as the substrate in a fed-batch SSF process, and the produced enzymes were used in LA production by *Bacillus coagulans* LA204. Because of the high capacity of inhibitors’ degradation by T*richoderma viride* R16 enzymes compared to some commercial ones the lactic acid production was increased by 24% (Liu et al., 2020).

Giacon et al. (2021) have studied the influence of two inhibitors furfural and HMF on the growth of five homofermentative (*Lactobacillus plantarum* CECT 221*, Lactobacillus delbrueckii, Lactobacillus plantarum* ESALQ 4*, Lactobacillus paracasei* LAB 4*, Lactobacillus paracasei* LAB 5) and seven (*Lactobacillus fermentum* DSM 20391*, Lactobacillus reuteri* ATCC 23272*, Lactobacillus fermentum* ESALQ 3, *Lactobacillus fermentum* ESALQ 5*, Lactobacillus fermentum* 1L-6-MRS*, Lactobacillus fermentum* 3L-2-M17, *Lactobacillus paracasei* LAB 2) heterofermentative lactic acid bacteria and have found that the effect of HMF and furfural on the growth rate of lactic acid bacteria (LAB) depended on the metabolic pathway. The growth kinetics in the presence of these compounds is enhanced for heterofermentative LAB, whereas is inhibitory to homofermentative LAB. The heterofermentative bacterium presented the ability to decrease the concentrations of furfural and HMF in the fermentation medium, with simultaneous lactic acid production. Low concentrations of these compounds present in the sugarcane bagasse hemicellulosic liquor did not have inhibitory effects on lactic acid production (Giacon et al., 2021).

Liu et al. (2013) compared the possibility for lactic acid production from glucose by three *Pediococcus* strains in the presence of inhibitors generated by acid hydrolysis of lignocellulosic biomass and reported that the strain *Pediococcus acidilactici* DQ2 could produce high concentrations of lactic acid from glucose in the presence of acetic acid, furfural, HMF, and vanillin in concentration range presented in acid pretreated biomass.

Oliveira et al. (2018) investigated the lactic acid production from sugarcane bagasse hydrolysates by *Lactobacillus plantarum* in the presence of furfural and HMF. The strain was capable to assimilate the inhibitors simultaneously with lactic acid production. A decrease of 86% for HMF and 98% for furfural was observed, together with 34.5 g/L lactic acid production. This approach could decrease the cost of the process eliminating the need for detoxification before fermentation (Oliveira et al., 2018).

Zhang et al. (2016) identified the inhibitory compounds generated during corn stover and corn cob pretreatment and investigated the toxicity limits of individual chemicals in the fermentation of hydrolysates to lactic acid by *Rhizopus oryzae*. They found that HMF and furfural were toxic at 0.5–1.0 g/L, while the carboxylic acid (formic, acetic, and levulinic) were non-toxic at concentrations less than 4 and 10 g/L. Among the phenolic *trans*-cinnamic acid and syringaldehyde had the highest toxicity at 1 g/L, while ferulic, *p*-coumaric, and syringic acids were not toxic. Although the concentrations in the hydrolysates were much lower than the toxicity levels of individual inhibitors, the lactic acid fermentation was considerably affected, suggesting possible synergistic action. The authors observed that while cell growth, lactate dehydrogenase, and lactic acid production were strongly inhibited, alcohol dehydrogenase and ethanol production were less or not affected. For the first time, this study showed that the inhibitors shifted the metabolic pathway from lactic acid to ethanol biosynthesis (Zhang et al., 2016).

Kunasundari et al. (2017) studied lactic acid production from oil palm sap by *B. coagulans* 191 strain and reported 53% conversion in case of non-treated sap, 54–74% when the sap was treated with charcoal, and up to 88% by acid and 92% for alkaline precipitation.

Cubas-Cano et al. (2020) investigated lactic acid production by three *B. coagulans* strains in defined media with inhibitors mixtures at high concentrations and hemicellulosic gardening hydrolysate pretreated by steam explosion. One of the isolates (A162) demonstrated high lactic acid productivity (up to 2.4 g L^−1^ h^−1^), even in presence of 5 g L^−1^ of furans and phenols (Cubas-Cano et al., 2020).

Zhang et al. used dry acid pretreated corn stover for LA production. The hydrolysate was neutralized by 20% Ca(OH)_2_ and bio-detoxified by solid-state fermentation with *Amorphotheca resinae* ZN1. The furfural and HMF content were fully degraded, and an increase of lactic acid production in simultaneous saccharification and co-fermentation (SSCF) of corn stover hydrolysate was observed (Han et al., 2018).

## 6 Microorganisms

Different groups of microorganisms (bacteria, fungi, yeasts, and algae) are capable to produce lactic acid from various substrates. Although many bacteria have been extensively used for lactic acid production as a primary or secondary end-product, the term lactic acid bacteria (LAB) is used for several genera. The recently reclassified genus *Lactobacillus* (into 25 new genera) (Zheng et al., 2020)*, Lactococcus, Enterococcus*, and *Pediococcus*, have been proven as the main producers. LAB are divided into two main groups - homo - and heterofermentative strains. Homofermentative LAB operate via Embden-Meyerhof-Parnas (EMP) pathway, expressing the aldolase enzyme with LA being the major product. They convert one molecule of glucose to two molecules of LA generating two molecules of ATP (Grewal et al., 2020). Some homofermentative strains metabolize sugars via the pentose phosphate (PP) pathway, producing 1.67 molecules LA from pentoses and hexoses and generating either 1.67 molecules (from hexose) or 0.67 (from pentose) ATP. Hetero-fermentative LAB are classified as obligate and facultative. Obligate LAB use exclusively the phosphoketolase (PK) pathway to convert one molecule of glucose or xylose to one molecule of LA and one molecule of ethanol or acetic acid and generate one molecule of ATP. Facultative heterofermentative LAB convert hexoses *via* EMP pathway and pentose *via* PK pathway (Cubas-Cano et al., 2018). Homofermentative strains producing optically pure L (+) or D (-) LA are preferred for industrial application due to the higher yield and easier downstream processing (Juturu and Wu 2016). Bacteria of genus *Bacillus* sp. (*B. subtilis, B. coagulans B. stearothermophilus, B. licheniformis, B. licheniformis*), *Corynebacterium glutamicum*, and *Escherichia coli* are also capable to produce lactic acid. Filamentous fungi are another important microbial producer of LA. Various representatives of the genus *Rhizopus* (*R. oryzae and R. arrhizus*) are utilized in free and immobilized form. The main advantages of using fungi for LA production are their amylolytic properties, simple medium requirements, their facilitate separation from the fermentation broth, and capacity to assimilate complex substrates like different lignocellulosic biomass. Numerous yeasts also can produce LA. Yeasts can tolerate low pH in comparison with LABs and thus minimizing the use of the neutralizing agent. The main disadvantage of yeasts is the low final LA concentration, but the use of engineered yeasts can overcome this drawback. The most important representatives of LA producing yeasts are *Candida* sp. (*C. sonorensis, C. boidinii,* and *C. utilise*), *Kluyveromyces* sp. (*K. lactis, K. marxianus*), *Pichia* sp. (*P. stipidis, P. pastoris*) as well as *Saccharomyces cerevisiae*. Other LA-producing species are algae and cyanobacteria. They are photosynthetic microorganisms, which can grow at minimal feed medium almost anywhere with a short growth cycle.

In a detailed review paper, Abedi and Hashemi listed almost all LA producing microorganisms together with their substrates, final LA concentration, LA yield, and productivity (Abedi and Hashemi 2020).

In Table 4 microorganisms capable of lactic acid production from lignocellulosic materials are summarized.

**TABLE 4:** Microorganisms, substrates, and lactic acid yield and productivity from different feedstocks.

Microorganism	Substrate	Optical isomer	Process organization	Lactic acid, g/l	Yield, g/g sugar	Productivity, g/l.h	Reference
*Lactobacillus plantarum* 23	*Chlorella vulgaris* ESP-31 hydrolysate	L (+)-LA	Batch	42.34	0.93	7.56	Chen et al. (2020)
			Continuous	39.72	0.99	9.93	
*Lactobacillus pentosus* ATCC 8041	Sugarcane bagasse	N/D	SSCF	65.0	0.93	1.01	Wischral et al. (2019)
*Lactobacillus pentosus CECT- 4023T (ATCC-8041)*	Sugarcane bagasse	N/D	Batch	55.44	0.72	0.43	González-Leos et al. (2020)
*Lactobacillus pentosus CECT 4023 T*	Wheat straw hydrolysate	N/D	Batch	12.58	0.55	0.22	Cubas-Cano et al. (2019)
*Lactobacillus pentosus (TBRC)*	Sugarcane bagasse	N/D	Fed-batch SSF	72.75	0.61	1.01	Unrean (2018)
*Lactobacillus pentosus FL0421*	Corn stover	N/D	Fed-batch SSF	92.30	0.66	1.92	Hu et al. (2016)
*Lactobacillus sakei* 25, *Weissella paramesenteroides* 24	40 g/L Green algae *Ulwa* sp. acid hydrolysate	N/D	Batch	25.14	0.78	6.79	Nagarajan et al. (2022)
*Lactobacillus plantarum* 23				24.98	0.73	6.25	
*Lactobacillus rhamnosus*				28.79	0.81	7.20	
				30.93	0.85	7.53	
*Lactobacillus sakei 25, Lactobacillus plantarum 23, Lactobacillus rhamnosus, Weissella* sp. *28, Weissella paramesenteroides 24 Weissella cibaria 27*	40 g/L Red algae *Gracilaria* sp. acid hydrolysate	N/D	Batch	28.45	0.84	3.56	Nagarajan et al. (2022)
				31.49	0.80	3.93	
				33.82	0.83	4.23	
				22.50	0.79	1.88	
				32.12	0.90	4.39	
				27.15	0.89	2.26	
*Lactobacillus sakei 25, Weissella paramesenteroides 24, Lactobacillus plantarum 23, Lactobacillus rhamnosus, Weissella cibaria 27, Weissella* sp. *28*	15 g/L Brown algae *Sargassum cristaefolium* acid hydrolysate	N/D	Batch	10.97	0.87	2.19	Nagarajan et al. (2022)
				10.38	0.87	2.08	
				11.65	0.89	2.53	
				10.80	0.88	2.15	
				9.90	0.86	1.65	
				9.02	0.83	1.51	
*Lactobacillus rhamnosus LA-04–1*	Cassava bagasse enzyme hydrolysate	N/D	SSCF	31.0	0.94	1.94	Chen et al. (2020)
*Bacillus coagulans LA-15–2*				30.0	0.91	1.50	
mixed culture				112.5	0.88	2.74	
*Lactobacillus rhamnosus ATCC 7469*	Forest and marginal lands lignocellulosic biomass	L (+)-LA	SHF	57.8	1.0	0.81	Pontes et al. (2021)
			SSF	61.7	1.0	1.4	
*Lactobacillus rhamnosus ATCC 7469*	Pressed recycled paper sludge enzyme hydrolysate	N/D	SHF	63.5	0.74	0.38	Marques et al. (2017)
			SSF	73.2	0.76	0.44	
			Pulsed SSF	108.2	0.62	0.9	
*Lactobacillus acidophilus BCRC 10695 and Lactobacillus plantarum BCRC 12327*	*Microalgae Gracilaria* sp. HCl and enzyme hydrolysate	N/D	Batch	19.32	0.65	-	Lin et al. (2020)
*Lactobacillus* sp. *L47*	Corn straw hydrolysate	L (+)-LA	pH controlled batch	99.8	0.67	-	Si et al. (2020)
*Lactobacillus* sp. *TERI-D3*	Rice straw hydrolysate	N/D	Batch	11.16	0.96	-	Verma and Subudhi (2021)
*Lactobacillus plantarum 23*	*Chlorella vulgaris* ESP-31 acid hydrolysate	N/D	Batch	40.30	0.97	6.72	Nandini et al. (2020)
			Continuous	37.76	0.91	12.59	
*Lactobacillus plantarum*	Rice straw hydrolysate	N/D	SSF	65.6	0.38	0.45	Tu et al. (2019)
*Lactobacillus rhamnosus ATCC 7469*	Brewer’s spent grain hydrolysate	L (+)-LA	pH-controlled batch	39.38	0.91	1.69	Pejin et al. (2017)
*Lactobacillus rhamnosus ATCC 7469*	Brewer’s spent grain hydrolysate	L (+)-LA	Batch	28.43	0.93	1.04	Radosavljevic et al. (2020)
*Lactobacillus rhamnosus ATCC 7469*	Waste bread stillage	L (+)-LA	Batch	50.59	0.91	1.40	Djukic-Vukovic et al. (2016)
	Waste potato stillage			46.21	0.81	1.28	
	Brewer’s spent grain hydrolysate			17.22	0.34	0.48	
*Lactobacillus rhamnosus CCM 1825*	Spent coffee grounds hydrolysate	N/D	Batch	25.69	0.98	0.35	Hudeckova et al., (2018)
*Lactobacillus rhamnosus + Lactobacillus brevis*	Sugar palm trunk pretreated enzyme hydrolysate	N/D	Batch	*33.29*	-	0.69	Erliana et al. (2020)
*Lactobacillus casei ATCC 393*	Distillers’ dried grains with solubles	L (+)-LA	Batch	101.7	0.84	3.9	Krull et al. (2020)
*Lactobacillus rhamnosus ATCC 10863*	Spent coffee grounds hydrolysate	N/D	SHF	24.95	0.91	0.59	Koo et al. (2019)
*Lactobacillus parabuchneri ATCC 49374*	Spent coffee grounds hydrolysate	N/D	SHF	6.5	0.56	0.54	Lee et al. (2021)
*Lactobacillus brevis ATCC 8287*	Spent coffee grounds hydrolysate	N/D	SHF	4.6	0.40	0.38	Lee et al. (2021)
*Lactobacillus delbrueckii ssp. delbrueckii CECT286*	Orange peel wastes hydrolysate	D (-)-LA	SHF	-	0.95	6.72	de la Torre et al. (2019)
*Lactobacillus delbrueckii ssp. delbrueckii CECT286*	Orange peel wastes hydrolysate	D (-)-LA	Batch				de la Torre et al. (2020)
			Resting cells	81.5	0.76	2.60	
			Growing cells	99.8	0.83	1.57	
*Lactobacillus. delbrueckii ssp. delbrueckii CECT 286*	Orange peel wastes hydrolysate	D (-)-LA	SHF	45	0.86	0.63	Bustamante et al. (2020)
*Lactobacillus. delbrueckii ssp. bulgaricus CECT 5037*	Orange peel wastes hydrolysate	D (-)-LA	SHF	39	0.84	0.55	Bustamante et al. (2020)
*Lactobacillus delbrueckii subsp. lactis*	Date palm waste hydrolysate	N/D	SHF	27.8	0.76	0.39	Alrumman (2016)
*Lactobacillus coryniformis*	Alkali pretreated DDGS	D (-)-LA	SHF	24.1	0.73	1.3	Zaini et al. (2019)
			SSF	27.9	0.85	1.5	
			Fed-batch SSF(11 g/L)	34.0	0.76	0.7	
			SSF(22 g/L)	38.1	0.70	0.8	
*Lactobacillus coryniformis subsp. torquens*	Pulp mill residue enzyme hydrolysate	D (-)-LA	Batch SHF	57	0.97	2.8	Moraes et al. (2016)
*Sporolactobacillus inulinus DSM 20,348*	Corn gluten acid hydrolysate	D (-)-LA	Fed batch	81	-	3.85	Brock et al. (2019)
	DDGS acid hydrolysate			107	-	3.44	
	Sunflower meal acid hydrolysate			103	-	3.27	
	Rapeseed meal enzyme hydrolysate			221	0.96	1.55	
*Bacillus coagulans*	Paper mill sludge	L (+)-LA	SSCF	82.6	0.83	0.69	Li et al. (2021)
*Bacillus coagulans ATCC 7050*	Macroalgae *Eucheuma denticulatum*	L (+)-LA	SSF	14.0	0.99	-	Chai et al. (2021)
*Bacillus coagulans NCIM 5648*	Alkali and enzyme pretreated sugarcane bagasse	L (+)-LA	SHF	69.2	0.55	2.88	Baral et al. (2020)
*Bacillus coagulans DSM ID 14–300*	Concentrated sugarcane bagasse HCl hydrolysate	L (+)-LA	Batch	55.99	0.87	1.7 g	Oliveira et al. (2019)
*Bacillus coagulans CC17*	Bagasse sulfite pulp enzyme hydrolysate	L (+)-LA	SHF	32.22	0.5	-	Zhou et al. (2016)
			SSF	50.20	0.84		
			Fed-bach SSF	110	0.72		
*Bacillus coagulans CC17A*	Wheat straw dilute acid and enzyme hydrolysate	L (+)-LA	SSCF	26.30	0.71	0.25	Ouyang et al. (2020)
*Bacillus coagulans LA1507*	Sweet sorghum bagasse, NaOH hydrolysate	L (+)-LA	SSF	111	0.73	1.59	Wang et al. (2016)
*Bacillus coagulans NCIM 5648*	Alkali and enzyme pretreated sugarcane bagasse	L (+)-LA	SHF				Nalawade et al. (2020)
			54.7 g/L glucose	50.4	0.92	2.4	
			62.7 g/L glucose	51.24	0.81	1.75	
*Bacillus coagulans AD*	Corn stover—wet explosion and enzyme hydrolysis	L (+)-LA	Continous	22.3–35.2	0.95	3.69	Ahring et al. (2016)
*Bacillus coagulans NBRC 12714*	Corn stover hydrolysate	L (+)-LA	Batch	98.3	0.95	3.28	Ma et al. (2016)
			Repeated batch	93.8	0.93	3.80	
			Continous	92.0	0.92	13.8	
*Bacillus coagulans strain H-1*	Corncob hydrolysate	L (+)-LA	Batch	68.0–36 h	0.85	1.8	Jiang et al. (2019)
				79.1–84 h	0.76	0.94	
*Bacillus coagulans LA-15–2*	Rice straw hydrolysate	L (+)-LA	Batch	63.5	0.3	3.18	Chen et al. (2019)
			Fed-batch	92.50	0.58	2.01	
*Bacillus coagulans*	Coffee mucilage	L (+)-LA	Batch	45.3 with yeast extract	0.77	4.4	Neu et al. (2016)
				43.3 without yeast extract	0.70	1.5	
*Bacillus coagulans*	Pretreated corn cob	N/D	Batch	77.34	0.82	1.61	Alhafiz et al. (2020)
*Bacillus coagulans JCM 2258 and JCM 9076*	Alkaline peroxide pretreated corn stover	L (+)-LA	SSF	-	0.33	-	Akao et al. (2016)
*Bacillus coagulans GKN316*	Corn stover diluted acid hydrolysate	L (+)-LA	Batch	35.37	0.83	0.91	Jiang et al. (2016)
	Acid-catalyzed steam-exploded hydrolysate			45.39			
	Acid-catalyzed			16.83			
	liquid hot water hydrolysate						
	Acid-catalyzed sulfite hydrolysate			18.71			
*Bacillus coagulans LA204*	NH_3_-H_2_O_2_ pretreated corn stover	N/D	Batch SSF	33.62	0.42	0.23	Liu et al., (2020)
			Fed-batch SSF	64.95	0.54	0.57	
*Bacillus coagulans 14–300*	Rye straw (RS) Digestate of energy corn silage hydrolysates (DCS)		SHF			1.73	Schroedter et al. (2021)
			RS	31.1	0.84	1.72	
			DCS	21.1	0.84		
			SSF				
			RS	39.3	-	0.82	
			DCS	15.7	-	0.58	
*Bacillus coagulans A-35*	Sweet sorghum juice	L (+)-LA	Batch				Olszewska-Widdrat et al. (2019)
			Lab. scale	78.75	0.78	1.77	
			Pilot plant	73.0	0.70	1.47	
*Pediococcus acidilactici ZY271*	Detoxified dry acid pretreated corn stover enzyme hydrolysate	L (+)-LA	Batch	61.6	0.87	2.34	Han et al. (2019)
*Pediococcus acidilactici PA204*	NaOH pretreated corn stover	N/D	Fed-batch SSF	25.92 (4% stover)	0.65	0.54	Zhang et al. (2020)
				92.01 (12% stover)	0.77	1.28	
				104.11 (15% stover)	0.69	1.24	
*Pediococcus acidilactici ZY271*	H_2_SO_4_ pretreated corn stover	L (+)-LA	SSCF with *Amorphotheca resinae* ZN1	130.3	-	1.81	Wei et al. (2018)
*Pediococcus acidilactici ZY15*	H_2_SO_4_ pretreated corn stover	D (-)-LA	SSCF with *Amorphotheca resinae* ZN1	124.8		1.73	Wei et al. (2018)
*Bacillus coagulans L-LA 1507*	NaOH pretreated corn stover	L (+)-LA	Fed-batch SSF	92.5	0.39	1.25	Chen et al. (2020)
*Rhizopus oryzae NLX-M-1*	XOS waste residue d from alkali-pretreated corncobs	L (+)-LA	SHF	34.0	0.34	0.71	Zhang et al. (2016)
			SSF	60.3	0.6	1.0	
*Rhizopus oryzae 3.819*	*Sophora flavescens* root	N/D	SHF	30.6	-	0.23	Ma et al. (2020)
			SSF	46.8	0.33	0.97	
*Rhizopus oryzae MTCC5384*	*Paper sldge*	N/D	SSF	27.0	0.36	1.19	Dhandapani et al. (2021)
*Escherichia coli JU15*	*Arthrospira platensis* hydrolysate	D (-)-LA	SHF	25.5	0.86	2.8	Werlang et al. (2020)
*Escherichia coli JU15*	Cassava bagasse hydrolysate	D (-)-LA	Batch	57.8	1.11	0.98	Utrilla et al. (2016)
*Escherichia coli AV03*	Corn stover hydrolysate			65.2	1.11	1.21	
*Enterococcus hirae ds10*	Sugar beet molasse	N/D	Batch	36.79	0.91	1.02	Abdel-Rahman et al. (2021)
			High cell density batch	49.49	0.91	0.41	
			fed-batch	61.76	0.97	2.06	
*Enterococcus mundtii DSM 4838*	Spent sulfite liquor	L (+)-LA	Fed-batch	87.9	1.0	3.25	Hoheneder et al. (2021)
*Enterococcus (97.6%)* dominated microbial consortia	Acid pretreated corn stover	N/D	SSF	43.73	0.50	0.32	Sun et al. (2021)
*Saccharomyces cerevisiae*	Spent coffee grounds	N/D	SSF	11.15	0.11	0.46	Kim et al. (2019)
*Trichoderma reesei/Lactobcillus pentosus*	Steam pretreated beech wood	N/D	SSCF	19.8	0.83	0.1	Shahab et al. (2018)

SHF, separate hydrolysis and fermentation; SSF, simultaneous saccharification and fermentation; SSCF, simultaneous saccharification and co-fermentation; N/D—not described.

### 6.1 Metabolic Engineered Microorganisms for High Lactic Acid Production

Normally, lactobacilii can not ferment pentose sugars, which is an obstacle to the effective utilization of hemicellulosic hydrolysates as a substrate. On the one hand, some of the highly productive strains produce a racemic mixture of D- and L-lactic acid. On the other hand, in hetero-fermentative lactic acid production, by-products are formed, which decrease the effectiveness of the process and increase the cost of pure lactic acid production. Metabolic engineering is a powerful tool finding increased application for overcoming some bottlenecks in lactic acid production from lignocellulosic biomass—low yield, substrate specificity, optical purity, acid tolerance, etc. Obtaining genetically modified strains by adding genes for pentoses assimilation, deleting undesirable branches in the metabolic pathway, or deleting one of the lactate dehydrogenase genes will help in using lignocellulosic feedstocks for pure lactic acid isomers production. Wu et al. (2021) summarized the advances in techniques of genome manipulation for engineering lactobacilli and future development of genetic tools for obtaining recombinant lactic acid bacteria.

The main approaches for obtaining high-performing strains LAB are adaptive evolution, mutagenesis screening, and metabolic engineering. Each of these methods has its own advantages and disadvantages. High-throughput screening techniques can be introduced into the post-mutagenesis screening process, but still relies heavily on random mutation and is time-consuming. In contrast, both metabolic engineering and adaptive evolution approaches targeted compensating shortcomings of mutagenesis screening. Currently, commonly used methods for the gene editing and metabolic engineering of LAB are plasmid-based homologous recombination, Red/RecET-mediated double-stranded DNA recombination, and single-stranded DNA recombination. Recently emerged CRISPR/Cas9 gene-editing technique is characterized by simple operation, efficiency, and precision (Tian et al., 2021). The gene manipulation tools of lactic acid bacteria are also discussed in Borner et al. (2019), Plavec and Berlec (2020), and Cho et al. (2020).

Upadhyaya et al. (2014) highlighted four main challenges (purity of lactic acid, acid tolerance of lactic acid bacteria, carbon sources, and parameters for industrial production) and summarized metabolic engineering solutions for lactic acid production.

Mazzoli (2020) outlined two main strategies in metabolic engineering of microorganisms for consolidated lactic acid production from lignocellulosic biomass—recombinant cellulolytic strategy, consisting in introducing cellulase systems in native producers of LA, and native cellulolytic strategy, aiming at improving LA production in natural cellulolytic microorganisms.

Cubas-Cano et al. (2019) applied adaptive laboratory evolution to improve the xylose fermentation capacity of a *Lactobacillus pentosus* strain. In sequential batch cultivation the ratio xylose: glucose was increased gradually from 15: 5 to 20: 0. When an improvement in bacterial growth and xylose consumption was detected the xylose content in media was increased. Clones were isolated from the final population of cells at different working conditions and that showed best performance was selected for further experiments. The selected strain (MAX2) showed up to 2-fold more xylose consumption and lactic acid production in comparison with the parental strain. The strain possessed high acidic tolerance and was capable to convert mixed sugars presented in a wheat straw hydrolysate (Cubas-Cano et al., 2019).

Other microorganisms were also been engineered for high lactic acid production. Yang et al. (2013) engineered *Thermoanaerobacterium aotearoense* by blocking the acetic acid formation pathway. The observed maximum l-lactic acid yield with the engineered strain was 0.93 g/g glucose, 0.79 g/g xylose, and 0.32 g/g xylan as a sole carbon source without any pretreatment. The obtained optical purity of l-lactic acid was 99.3% and the engineered strain was capable of high production in non-sterile fermentation (Yang et al., 2013).

Kong et al. (2019) described the genetic manipulation of a yeast strain *Kluyveromyces marxianus* for effective LA production from corncob. Introduction of heterologous lactate dehydrogenase gene (from *Plasmodium falciparum* or *Bacillus subtilis*) and the proton-coupled monocarboxylate transporter gene from *Saccharomyces cerevisiae* together with disruption of putative d-lactate dehydrogenase in *Kluyveromyces marxianus* led to production of 103.00 g/l l-lactic acid with optical purity of 99.5% from 180.00 g/L corncob residue *via* simultaneous saccharification and co-fermentation (Kong et al., 2019).

Kuo et al. (2015) isolated twenty-six strains from soil and putrid fruits and tested the ability of 11 of them to ferment wood hydrolysate. *Lactobacillus paracasei* 7B showed high lactic acid productivity and high tolerance to inhibitors was chosen for further gene manipulation by interruption of *ldhD* gene. The resulting strain was able to ferment glucose and lignocellulosic hydrolysates of wood and rice straw without detoxification and achieved high yields—215 g/L in fed-batch glucose fermentation, 99 g/L from the undetoxified and 102 g/L from the detoxified hydrolysate (Kuo et al., 2015).

Turner et al. (2016) constructed a recombinant *Saccharomyces cerevisiae* strain capable of assimilating cellobiose and xylose and producing lactic acid. Different genes (*cdt-1, gh1-1, XYL1, XYL2, XYL3*, and *ldhA*) coding cellobiose transporter, β-glucosidase, xylose reductase, xylitol dehydrogenase, xylulokinase, and lactate dehydrogenase were integrated into the *S. serevisiae*. In the engineered strain native pyruvate decarboxylase (*pdc*) and alcohol dehydrogenase (*adh*) genes were not deleted, but still, almost no ethanol was produced when fermenting a cellobiose and xylose mixture. The engineered strain produced 83 g/L of lactic acid with a yield of 0.66 g/g sugar from a cellulosic sugar mixture (10 g/L glucose, 40 g/L xylose, and 80 g/L cellobiose) (Turner et al., 2016).

Kim et al. (2019) engineered *Saccharomyces cerevisiae* SR8 to produce lactic acid and ethanol from pretreated spent coffee grounds. The pRS41N-Cas9 plasmid was introduced and expression of the lactate dehydrogenase gene was confirmed (Kim et al., 2019).

Using helium-based atmospheric and room temperature plasma mutation and evolution Jiang et al. (2016) adapted *Bacillus coagulans* NL01 strain to overcome the inhibitors’ action in corn stover hydrolysates. The mutant strain *Bacillus coagulans* GKN316 was capable to convert different hydrolysates to lactic acid with high inhibitor tolerance. The individual inhibitory effect of furfural, 5-hydroxymethylfurfural, vanillin, syringaldehyde, and *p*-hydroxybenzaldehyde was also studied and was found that the syringyl compound was most toxic. The strain *B. coagulans* GKN316 could effectively convert these inhibitors to the less toxic corresponding alcohols (Jiang et al., 2016).

Most of the microorganisms producing lactic acid ferment sugars to l-lactic acid and there are only a few capable to produce d-lactic acid. In recent years, the demand for d-lactic acid has increased, especially for the production of polylactic blends (stereo-complex polylactic acid, composed of both L and d-lactic acid) which is characterized by a high melting point. Therefore, the interest in d-lactic acid-producing strains, including genetically manipulated also increases.

Zhang et al. investigated d-lactic production from glucose, xylose, and alkaline corn stover hydrolysate by an l-lactate deficient strain of *Lactobacillus plantarum* in which xylose-assimilating genes encoding xylose isomerase and xylulokinase were successfully cloned. The engineered strain produced 19,7 g/l d-lactic from 40 g/L xylose in a batch experiment. In a fed-batch mode production of 30.1 g/L of d-lactic was achieved. When a mixture of glucose and xylose (2:1 ratio) was used as a substrate, 47.2 g/l d-lactic was produced, with a lactic acid yield of 0.84 g/g. The experiments with corn stover were carried out in two modes—separate saccharification and fermentation (SHF) and simultaneous saccharification and fermentation (SSF). In the first case, 19.4 g/l d-lactic acid was produced, while in the second—26.8 g/L when the hydrolysate was supplemented with yeast extract and 29.4 g/L when soybean meal extract was added. Finally, in a fed-batch experiment with corn stover hydrolysate in SSF mode production of 61.3 g/l d-lactic acid was achieved. The production of acetic acid was observed in all experiments (Zhang et al., 2016). In other work, the authors constructed and compared an l-lactate-deficient mutant strain *Lactobacillus plantarum* NCIMB 8826 *ldhL1* and its derivative harboring a xylose assimilation plasmid (*DldhL1-pCU-PxylAB*). Recombinant *DldhL1-pCU-PxylAB* used xylose to produce high yields of d-lactic acid and was able to ferment xylose and glucose simultaneously which is an important advantage when using lignocellulosic biomass as a substrate for producing lactic acid. As substrates for d-lactic acid production, corn stover and sorghum stalk hydrolysates were successfully used. *DldhL1-pCUPxylAB* produces 20% more d-lactic acid than *DldhL1* from lignocellulosic biomass. In the SSCF process the yield increased about 38%, and productivity—almost three-fold when compared with SHF process (Zhang et al., 2016).

In a series of three papers, Kondo and coworkers studied the possibility of d-lactic production with engineered *Lactobacillus plantarum* strains. Firstly, they engineered an l-lactate dehydrogenase-deficient *Lactobacillus plantarum* strain for d-lactic acid production from cellulosic materials. When an endoglucanase-secreting plasmid was introduced into *L. plantarum* 1.27 g/l d-lactic acid was produced with 2 g/L cellohexaose as substrate and 1.47 g/L with barley β-glucan as substrate. Although anaerobic conditions partially suppressed this conversion of d-lactic to acetic acid, the final product was predominantly acetic acid (Okano et al., 2010). In another paper, the xylose assimilating operon from *Lactobacillus pentosus* was introduced into an l-lactate dehydrogenase deficient *Lactobacillus plantarum* to achieve efficient d-lactic acid fermentation from a mixture of xylose and glucose. Successful homo-d-lactic acid production was achieved 41.0 g/L lactic acid (88% g/g xylose yield and 98.7% optical purity) from 50 g/L xylose. In the case of a mixture of xylose and glucose (1:3 ratio) 74.2 g/L of lactic acid was produced with 0.78 g/g sugar yield with d-lactic acid optical purity of 99.5%. Finally, a mixture of three sugars—xylose, glucose, and arabinose (5:10:1 ratio) and 61.2 g/L of lactic acid was produced with a yield of 0.80 g/g sugar consumed and 99.5% optical purity was produced from 80 g/L sugars. Simultaneous sugars utilization was achieved without carbon catabolite repression (Yoshida et al., 2011). The last paper was focused on the d-lactic acid production from delignified hardwood pulp. The previously described *Lactobacillus plantarum* mutant (Yoshida et al., 2011) was used for d-lactic acid production from both glucose and xylose in a simultaneous saccharification and fermentation (SSF) process. The SSF resulted in 55.2–84.6 g/L lactic acid, depending on the load (5–15%) after 72 h. To improve the enzymatic saccharification at high-load, short-term pulverization of pulp was conducted. The pretreatment significantly ameliorated saccharification and suppressed the formic acid by-product formation. In this case, SSF resulted in a lactic acid production of 102.3 g/(0.88 g/g-sugars yield) and optical purity of 99.2%. (Hama et al., 2015).

Žunar et al. (2020) manipulated a wild strain *Lactobacillus gasseri* JCM 1131T firstly by transforming with plasmids carrying additional copies for each of the three lactate dehydrogenases that the wild type encodes for. Secondly, each of the three endogenous genes for lactate dehydrogenases was inactivated using the plasmid *pHBintE,* which was used for the first time to inactivate genes in lactobacilli. Transformation of *L. gasseri* with plasmids carrying additional genes for L- or d-lactate dehydrogenases didn’t affect the ratio of produced stereoisomers, but inactivation of the endogenous genes created strains which yielded 0.96 g/g glucose of either L- or d-lactate. Constructed strains efficiently fermented wheat straw hydrolysate and produced 0.37–0.42 g of lactate/g wheat straw (Žunar et al., 2020).

Prasad et al. (2020) described the application of an evolutionary engineered thermo-tolerant strain of *Lactobacillus bulgaricus* strain in d-lactic acid production from rice straw. The engineered strain was capable to work at 45°C and thus be used in an SSF process enzyme significantly reducing the enzyme loading. The powdered rice straw biomass was pretreated by dilute acid followed by steam explosion. The rice straw was soaked with 0.2% w/v H_2_SO_4_ for 24 h and then heated to 175°C for 30 min followed by rapid decompression to atmospheric pressure. The pretreated rice straw contained 45.84% cellulose and 4.63% xylan and was used in further experiments after enzymatic hydrolysis with commercial cellulase complex (SacchariSEB C6L Plus). SHF and batch and fed-bach SSF mode of fermentation were carried out. In batch SSF experiments 11.8–32.4 g/l d-lactic acid was produced, depending on the enzyme and total solid loading, while in fed-batch SSF the final obtained lactic acid concentration was 108.6 g/L (Prasad et al., 2020).

Tian et al. (2021) developed through the CRISPR-Cas9 gene-editing platform a high optical purity l-lactic acid producing strains. Further, by adaptive evolution a high-performance strain (NCBIO01-M2-*ldhL1-HT*) was obtained, that could efficiently produce l-lactic acid at a high temperature (45°C). The strain was capable of producing 221.0 g/L of l-lactic acid in open fermentation. The productivity and yield were above 7.5 g/L/h and 0.96 g/g respectively, and the optical purity of l-lactic acid exceeded 99.1% (Tian et al., 2021).

A lactogenic *Escherichia coli* strain JU15 (MG1655, *ΔpflB, ΔadhE, ΔfrdA, ΔxylFGH, gatC-S184L, ΔmidarpA*, and *Δreg 27.3* *kb*) was used and modified to produce d-lactic acid from sugarcane bagasse and corn stover hydrolysates. The strain JU15 was additionally genetically modified by deleting the pathways for the production of l-lactic acid and acetic acid, thus leading to a new strain - AV03 (JU15, *ΔpoxB, ΔackA-pta, ΔmgsA*). While JU15 showed sequential sugars assimilation and acetic acid production, AV03 showed simultaneous sugars consumption no acetic acid production, with a minimal nutrient addition in pH-controlled fermentation. The d-lactic acid yield in all cases was close to 0.95 g/g sugars (Utrilla et al., 2016).

Werlang et al. (2020) have also used the recombinant *Escherichia coli* strain JU15 for d-lactic acid production from algal biomass pretreated by acid and enzyme hydrolysis. The strain was capable of producing 25.5 g/L d-lactate (86% of the theoretical conversion of glucose to lactate) after 9 h of fermentation with 0.255 g/g of dried *A*. *platensis* biomass (Werlang et al., 2020).

### 6.2 Immobilized Microorganisms in Lactic Acid Production From Lignocellulosic Biomass

Cells immobilization offers undoubted advantages in a fermentation process such as the possibility of realization of fed-batch or continuous process; high cells concentration; increased stability of the biocatalyst and reduced influence of substrate and product inhibition; easier separation of biocatalyst from fermentation broth; and higher fermentation rate. Different immobilization methods were used in lactic acid production from biomass—adhesion, encapsulation, gel entrapment, etc.

Radosavljevic et al. (2021) used *L. rhamnosus* cells immobilized by encapsulation in polyvinyl alcohol cryogel for LA production from brewer’s spent grain (BSG) and malt rootlets (MR) hydrolysates. For hydrolysate production, dry BSG and MR in ratio 4:1 were sequentially mixed with Termamyl SC (amylase), SAN Super 240 L (glucoamylase), and Cellic CTec2 (cellulase) enzymes. After enzymatic hydrolysis, the obtained hydrolysate was separated from solids and vacuum concentrated to 5.5% reducing sugars content before was used in LA fermentations. Application of 10% PVA in BSG and MR hydrolysate throughout 12 consecutive batch fermentations resulted in high LA yield and volumetric productivity of 97.1% and 2.1 g/L h^−1^, respectively (Radosavljevic et al., 2021).

*Lactobacillus bifermentans* cells were immobilized in Ca-alginate gel for lactic acid production from the wheat bran hemicellulosic hydrolysate. Cells entrapped in calcium alginate beads can consume all glucose and arabinose and 75% of xylose in one step. The maximum values of lactic acid yield, productivity, and percent sugar utilization were 0.83 g/g, 1.17 g/l.h, and 76%, respectively, at temperature 42°C and pH 7.5 (Givry et al., 2008).

Mladenovic et al. (2018) have used *Lactobacillus paracasei* immobilized by adhesion onto agro-industrial residues—sunflower seed hull, brewer’s spent grain, and sugar beet pulp for lactic acid production from distillery waste potato stillage remaining after bioethanol production. The immobilized preparations were stable at least in five consecutive cycles and a maximal LA concentration of 80.1 g/L with an average yield coefficient of 0.97 g/g was achieved using sugar beet pulp. The LA productivity was 1.48 g/l.h. In a later paper (Mladenovic et al., 2019) extended their research on the possibility of simultaneous production of LA and probiotic enriched livestock feed on a combined substrate based on molasses and potato stillage. The highest total LA concentration of 399 g/L and overall productivity of 1.27 g/l.h was achieved in five repeated fermentation batches.

Shi et al. (2012) investigated the production of l-lactic acid by immobilizing *Lactococcus lactis* cells in a fibrous bed bioreactor system. The substrate was *Jerusalem artichoke* hydrolysate and the immobilization was done by adhesion in a column packed with spiral wound cotton towel. Using the fed-batch strategy, 142 g/L lactic acid was produced. Subsequent repeated-batch fermentations further exhibited the persistence and stability of the system for the high production of l-lactic acid in a long term.

*Lactobacillus plantarum* 23 immobilized in polyvinyl alcohol beads was tested for LA production from renewable feedstocks (Nagarajan et al., 2020). Sugarcane bagasse, cheese whey, and microalgal biomass of *Chlorella vulgaris* and *Ulva* sp. were used as substrates after pretreatment. Sugarcane bagasse was hydrolyzed by two steps—phosphoric acid hydrolysis to remove the lignin fraction and obtain bagasse cellulose, followed by sulfuric acid hydrolysis to hydrolyze the cellulose. Microalgal biomass was subjected to a combined acid/thermal pretreatment (4% H_2_SO_4_, 120°C, 20 min). Lactic acid fermentation was carried out in batch and continuous mode. The best results were obtained with sugarcane bagasse—41–42 g/L LA and 0.98–1.0 g/g sugars and microalgal biomass—37.9 g/L and 0.91 g/g sugars. In another publication (Chen et al., 2020) extended the research on LA production from *Chlorella vulgaris* biomass with PVA immobilized *Lactobacillus plantarum,* including *in-situ* LA removal by ion exchange. Optimal conditions of fermentation were determined and a substantial increase in LA yield (72%) was observed in the case fed-batch coupled with ion exchange. Continuous fermentation using immobilized *L. plantarum* with high productivity was demonstrated (14.22 g/l.h from glucose and 9.93 g/l.h from algal biomass) and immobilized beads could be used for 4 months without loss of activity.

Thakur et al. (2019) investigated lactic acid production from sugarcane molasses by calcium alginate immobilized *Lactobacillus casei* MTCC 1423 cells. Different process parameters (bead diameter, bead coating, biomass concentration, shaking speed, substrate concentration, nitrogen content, temperature, incubation time, and pH) were optimized. The immobilized cells were stable between 4 and 9 cycles depending on the bead coating method. At optimal conditions, 128.45 g/L lactic acid was achieved (Thakur et al., 2019).

## 7 Process Organization

All well-known biotechnological fermentation methods are also applicable in lactic acid fermentation from lignocellulosic and industrial wastes. Batch fermentation is the widely used and simplest method. The fermentation medium is seeded with the appropriate microorganism and no product is extracted or substrate is added to the end of the process. High final LA concentration and low risk of contamination are the main advantages of this fermentation mode, while low cell densities, low productivity, and substrate and/or product inhibition are the main disadvantages. The substrate inhibition can be overcome by fed-batch—substrate is added in portions and no product is withdrawn, but still, product inhibition is the major obstacle. In repeated batch (semicontinuous), fermentation a part or all of the cells from a previous run are inoculated into the next run. The semicontinuous mode can assure a decrease in time, energy costs, and increased LA productivity. Continuous fermentation is realized by steadily fresh medium feeding and fermentation broth withdrawal. The dilution rate (the velocity of substrate feeding and product withdrawal) must be carefully chosen, so no cell washing or product accumulation is accomplished. The main disadvantages of this process organization are incomplete substrates utilization and low LA concentration. High cell density fermentation using cell recycling is an efficient strategy for enhancing sugar consumption. The cells retention by ultrafiltration permits separation and recirculation. Cells immobilization can resolve the problem with retention of the cells into the system and facilitate continuous process realization. Different reactor types– continuous stirred tank, packed bad, and fibrous bad were utilized.

The necessity of individual sugars production by hydrolysis of cellulose and hemicellulose in pretreated lignocellulosic biomass leads to the use of enzymes like amylases and cellulases. Separate hydrolysis and fermentation (SHF) and simultaneous saccharification and fermentation (SSF) are the main strategies for lignocellulosic and waste biomass utilization. An asset of SHF is that hydrolysis and fermentation are separately performed under their optimal conditions. Usually, hydrolysis and fermentation have different optimal conditions (temperature and pH). The drawback is that during enzymatic hydrolysis, the sugars released might lead to an increase of the inhibition and suppress enzyme activity. On the other hand, SSF offers more advantages than SHF—the possibility of a one-pot process, reducing time and cost of fermentation, and reducing inhibition. The mentioned above difference in optimal temperatures and pH for both hydrolysis and fermentation is the main drawback of SSF. A possible way of overcoming this discrepancy lies in using microorganisms releasing cellulitic enzymes during so-called simultaneous saccharification and co-fermentation (SSCF). Tarraran and Mazzoli (2018) reviewed recent research in co-fermentation of LAB with native cellulolytic microorganisms, as well as the construction of recombinant cellulolytic LAB by metabolic engineering, for direct fermentation of plant biomass for reducing the high cost of exogenous cellulase supplementation. Zaini et al. (2019) compared SHF and SSF approaches for d-lactic acid production from DDGS by *Lactobacillus coryniformis*. They reported that SSF demonstrated better fermentation characteristics compared to SHF [Zaini, Chatzifragkou and Charalampopoulos, Microbial production of d-lactic acid from Dried Distillers Grains with Solubles (DDGS) 2019]. An additional plus offers the possibility of such microorganisms to grow in the presence of inhibitors released during pretreatment. Some of them use the inhibitors as a carbon source and thus decreasing their concentration and negative effect on LA fermentation. Examples of using different modes of fermentation are listed in Table 4.

Integration of fermentation and separation steps *in situ* extractive fermentation gives the possibility of increasing productivity and decreasing energy consumption. Different methods are proposed for lactic acid removal—ion exchange (Othman et al., 2018), membrane bioreactors (Gössi et al., 2020), liquid-liquid extraction (Lan et al., 2019). Although process integration is well documented in the case of lactic acid production from sugars (M. Othman et al., 2017), there are no published results for second-generation biomass.

Some advantages and drawbacks of different process organizations are presented in Table 5.

**TABLE 5:** Advantages and drawbacks of different process organizations.

Fermentation mode	Advantages	Disadvantages
Batch	Ease of operation; Low risk of contamination	Substrate and product inhibition
	High cell concentration	Low yield
Fed batch	Less substrate inhibition; Increase cell concentration	Product inhibition; Difficulties of maintaining process conditions
Repeated batch	Short process time; Increased cells growth	Decreasing productivity with increasing batch number; Problems with cells viability and stability
Continuous	Controlled growth; High productivity	Incomplete substrate utilization; Possible cells washing or product accumulation
Separate hydrolysis and fermentation	Each process is performed at optimal condition; Increased productivity; Low enzyme intake	Higher risk of contamination; Increased inhibition; Requires more equipment
Simultaneous saccharification and fermentation	Shorter time; Reduced reactor volume; Reduced inhibition; Low cost	Difficulties in matching of optimal conditions for both processes
Separate hydrolysis and co-fermentation	Bothe processes are performed at optimal conditions; Increased product yield	Increased risk of contamination; Increased enzyme requirements; Increased inhibition
Simultaneous saccharification and co-fermentation	Lower risk of contamination; Lower costs; Shorter time	Increased enzyme requirements; Difficulties in matching of optimal conditions for both processes

## 8 Separation

Downstream processing (separation, purification, and recovery) is a very important step in lactic acid production and its cost can reach up to 50% of the total price of the final product. In general, separation and purification methods of second-generation LA (from lignocellulosic feedstocks) don’t differ from these applied for first-generation LA. Having in mind that the use of pretreated biomass for LA production can result in a more complex fermentation broth, containing more impurities (unfermented pentoses and hexoses, inhibitory substances, lignin, protein, pigments, and salts), a more complex and expensive downstream process will be needed. Therefore, searching for a highly effective, improving LA yield, low-cost, and environmentally friendly method is of great importance. It is well known that LA fermentation is characterized by relatively low final concentration and low production rate, due on one hand, to the change of pH caused by acid accumulation and, on the other hand, to the product inhibition. Hence, neutralization of the broth or *in situ* removal of the product and thus increasing productivity is necessary. Sodium hydroxide, ammonia Ca(OH)_2_, or CaCO_3_ are used as neutralizing agents.

Conventionally, industrially produced by fermentation LA is removed by precipitation with Ca(OH)_2_. The method suffers from many drawbacks—precipitated calcium lactate has considerable water solubility [5–20%, depending on temperature (10–50°C)], additional steps have to be applied for converting lactate to free lactic acid—filtration, acidification with H_2_SO_4_ (generates a large amount of CaSO_4_), activated carbon purification and decolorization, concentration by evaporation and crystallization. In the case of lignocellulosic biomass-derived substrates, the separation process is additionally complicated by other simultaneously precipitated.

Because of numerous disadvantages of the precipitation method, different techniques have been investigated as an alternative to the traditional recovery process. These include liquid-liquid extraction (including liquid membranes), electrodialysis, ion exchange, membrane separation, aqueous two-phase systems, etc.

Liquid-liquid extraction is a separation method based on different solubility of the target compound in water and immiscible organic solvent (hydrocarbons or alcohols with high molecular mass). For increasing the partition a chemical called extractant is added to the organic phase. The target compound (lactic acid) forms a complex with the extractant with an increased affinity towards the organic phase.

Different membrane-based separation techniques (micro-, ultra-, nano-filtration, reverse osmose, and pervaporation) can be successfully used as a preliminary step for lactic acid purification. These methods are environmentally friendly and easily scaled up. Usually, they are employed for the separation of cells, proteins, and sugars.

Adsorption is a powerful method for bioseparation with easy regeneration, low energy consumption, and high selectivity. Neutral (activated carbon) or ion exchange adsorbents can be used. Din et al. (2021) analyzed in-depth second-generation lactic acid separation and recovery from fermentation broth by ion exchange. The authors highlighted different factors influencing the process and briefly discussed other methods for LA separation.

Electrodialysis is a method facilitating salt ions transport through an ion-exchange membrane from one to another solution by applying an electric field. Mono- and bipolar electrodialysis are widely used for lactic acid separation.

Aqueous two-phase systems (ATPS) are formed when two water-soluble polymers (or a polymer and a salt) are mixed in a common solution. Because of the incompatibility of the constituents, the system is separated into two phases. ATPS are extensively used in the separation of various bioproducts. Zhou et al. (2021) investigated the separation of lactic acid from simulated and actual lignocellulosic (from SSCF of dilute acid-pretreated corn stover) fermentation broth. In a three-stage ionic liquid-based sugaring-out extraction from the filtered and unfiltered fermentation broth, the total recovery of LA was 89.5 and 89.4%, respectively (Zhou et al., 2021).

Other methods like molecular distillation, short path evaporation, salting-out extraction, emulsion liquid membranes are also tested for lactic acid separation. There are numerous papers concerning lactic acid separation from the fermentation broth, but the majority of them are devoted to lactic acid produced of sugars or starchy materials (first generation LA), and data for the separation of lactic acid obtained from lignocellulosic biomass are scarce. Several review papers summarize recent progress in lactic acid separation by different methods (Kumar et al., 2019; Li et al., 2021; Yankov 2020). De Oliveira et al. (2020) discussed current state-of-the-art on the separation and purification methods of lactic acid derived from the fermentation of second-generation feedstocks.

Baral et al. (2021) investigated lactic acid separation from the broth after sugarcane bagasse hydrolysate fermentation. Various extractants (tripropylamine, trihexylamine, trioctylamine, triisootcylamine, and tributylphosphate) and diluents (chloroform, dodecane, ethyl acetate, MIBK, hexane, and 1-octanol) were screened for choosing the best extractant-diluent pair. Tributylphosphate and ethyl acetate were selected because of their higher extraction efficiency—59.63 ± 1.28%. Different salts—calcium chloride, ammonium sulfate, sodium dihydrogen phosphate, and,di-potassium hydrogen phosphate were used in the view to enhance extraction. Salting-out extraction with 60% ammonium sulfate increased the lactic acid extraction efficiency to 85.95 ± 0.44%. About 80% salt recovery was achieved using chilled acetone (Baral et al., 2021).

Demmelmayer and Kienberger (2022) studied reactive extraction of lactic acid from sweet sorghum silage press juice. Different extractants (dioctylamine, trioctylamine, and Aliquat 336) and diluents/modifiers (octanol, decanol, hexane, nonane, and undecane) were used in various ratios. Stripping of the loaded organic phase was done by sodium hydroxide. The extraction efficiency of 41.1% was reached using system DOA/ALIQ:1–octanol:n–nonane (35:35; 15:15) with a stripping efficiency of 98.2% (Demmelmayer and Kienberger 2022).

Hu et al. (2017) explored lactic acid recovery from fermentation broths of food waste and bakery waste hydrolysates by continuous ultrasonic solvent extraction. They compared the extraction efficiency of six organic solvents and chose ethyl acetate (EA) as the best option. The extraction into ethyl acetate (LA:EA ratio 1:2) by sonication at pH 2 under room temperature resulted in 82–84% yield and 98% purity (Hu et al., 2017).

Lan et al. (2019) compared two methods for lactic acid extraction from corn stover hemicellulose-derived liquor (HDL). In the first one, 10% trioctylamine in octanol was used with 50.8% extraction efficiency. An additional salting-out extraction step was included before TOA extraction. The salting-out extraction was done in THF/NaCl system. The overall efficiency of 83% was achieved in the two-step extraction of an activated carbon-treated HDL. The process provided stable LA removal during five consecutive extraction/striping cycles (Lan et al., 2019).

Xu et al. (2018) studied salting-out extraction of lactic acid in systems composed of Na_2_SO_4_ and organic solvent (1,4-dioxane, THF, γ-butyrolactone, and γ-valerolactone). Best results were obtained with the system Na_2_SO_4_/1,4-dioxane, and the conditions for LA extraction were further optimized. In the case of 13.5% Na_2_SO_4_/28% 1,4-dioxane, at low pH (1.68) 91% extraction efficiency was achieved in the case of model LA solution and 90% from real mixture directly derived from the chemical catalytic conversion of corn stover (Xu et al., 2018).

Chen et al. (2020) realized a combined process for LA production and separation. After fermentation of corn cob hydrolysate, the fermentation broth was fed into an ultrafiltration cell and retentate, containing mainly cellulase was recycled. The permeate was acidified to liberate free acid from calcium lactate and the liquid fraction was transferred in a nanofiltration unit. The permeate was further purified by distillation for pure lactic acid production, while the retentate, containing mainly residual sugars was transferred back in the fermentor. A total of six cycles of fed-batch SSF were performed (Chen et al., 2020).

Short-path evaporation of lactic acid from hemicellulose hydrolysate after acid hydrolysis of sugarcane bagasse was investigated by De Oliveira et al. (2020) During the fermentation pH of the broth was maintained by Ca(OH)_2_. At the end of fermentation, the broth was treated with sulphuric acid for calcium lactate destroying and then filtered and centrifuged. The clear liquid was evaporated and a concentration of 3 times (from 27.85 g/L to 86.69 g/L) was obtained at 120°C evaporator temperature of, 13°C condenser temperature of, and 8.27 ml/min feed flow rate. The authors mentioned a more difficult process than the separation of LA from sugars (Oliveira et al., 2019).

Garett et al. (2015) realized an *in situ* extractive fermentation of lactic acid from corn stover sugars. The corn stover was pretreated by a wet explosion followed by enzyme hydrolysis. The resulting sugar solution was fermented by a mixed culture, dominated by a *Bacillus coagulans* strain. A weak base resin Amberlite IRA-67 was used for LA removal during fermentation. The base eluted resin was able to maintain stable fermentation over 108-days, during which the average loading of lactic and acetic acids was 112.2 and 19.6 mg acid/g resin, respectively (Garrett et al., 2015).

Oonkhanond et al. (2017) used pretreated sugarcane bagasse in an integrated system with *ex-situ* nanofiltration. The first step fractionation was carried out using acid ethanolysis (5N H_2_SO_4_ in 50% v/v ethanol) at 90°C for 4 h. In the second step, the neutralized solid part was treated with an alkaline/peroxide reaction at 4.5% wt H_2_O_2_ at pH 11.5 for 24 h at 60°C. and finally was hydrolyzed by Accellerase 1,500 at pH 5.0, 50°C for 96 h. Using nanofiltration with a low flux membrane, lactic acid was efficiently separated from the fermentation broth, and after vacuum distillation, a lactic acid solution with 80% purity was achieved (Oonkhanond et al., 2017).

Alexandri et al. (2018) carried out a pilot-scale LA production using a defined medium with glucose, acid whey, sugar bread, and crust bread, coupled with membrane technologies. Microfiltration and nanofiltration were successfully used to separate lactic acid from the majority of the other fermentation components, leading to a more than 10% improvement of lactic acid’s purity (Alexandri et al., 2018).

Neu et al. (2016) explored different methods for downstream processing of a lactic acid stream obtained after fermentation of coffee mucilage. Micro- and nanofiltration, electro-dialysis, ion-exchange, and distillation result in a 930 g/L lactic acid solution with 99.8% purity (Neu et al., 2016).

Olszewska-Widdrat et al. (2019) investigated complex method combining various technics in LA separation from sweet sorghum juice—filtration and ultra-filtration, chelating resin, mono- and bipolar electrodialysis, followed by chromatography and vacuum evaporation were subsequently carried out producing a 905.8 g/L lactic acid solution, with an optical purity of 98.9% (Olszewska-Widdrat et al., 2019).

Prado-Rubio et al. (2020) presented a techno-economic assessment of lactic acid (produced from sugarcane feedstock) separation. The evaluation of three separation methods (reactive distillation, reactive extraction, and electrodialysis) was made based on a LA/water stream in mass ratio 0.3038/0.6962. The calculations were made using a specific methodology design for each separation technique applying Aspen Plus, Matlab, and their combination. Taking reactive distillation as a base, other methods showed a significant reduction in total annual cost—reactive extraction—44% and electrodialysis—55% (Prado-Rubio et al., 2020).

Mandegari et al. (2017) investigated four scenarios for ethanol and/or lactic acid production in lignocellulose biorefineries annexed to a typical sugar mill using Aspen Plus simulator. Economic evaluation, energy assessment, and environmental life cycle assessment (LCA) were carried out. The LCA suggested that all LA producing scenarios introduced environmental burdens (Mandegari et al., 2017).

## 9 Conclusion and Perspectives

In the way of decreasing dependence on petroleum-based production of valuable chemicals, considerable efforts are concentrated on the investigation of different lignocellulosic feedstocks as raw materials for fermentative production of various chemical products. Fermentative lactic acid production from first-generation biomass is an example of a growing production process. High cost of the substrates and enzymes, some environmental issues, food competition, and waste generation are the factors hindering the industrial process implementation. Second and third-generation sources present an attractive alternative for lactic acid production. From the summarized here research during the last 5 years, the most employed feedstock is corn stover and the most used pretreatment method—acid and enzyme hydrolysis. Nevertheless, there are numerous challenges in the route towards a cost-effective process—separation of lignin from cellulose and hemicellulose; release of inhibitory compounds during pretreatment; product and substrate inhibition; and by-product formation in fermentation; the more complex composition of fermentation broth in and ecological problems connected with classical precipitation separation, etc. However, there are some encouraging signs of developing an economically effective and environmentally friendly method for lactic acid production from renewable resources. First of all, is the progress in using green solvents like ionic liquids, capable of being more effective and producing fewer inhibitors during lignocellulose dissolution. What is more, ionic liquids have very tunable characteristics and can be easily tailored for different feedstocks. Secondly, developing genetically modified microorganisms with high productivity and adapted to inhibitors. Finally, process integration—elaboration of different SSF processes with different microorganisms, as well as *in situ* extraction, both leading to minimization of the substrate and product inhibition and thus increasing the yield and decreasing the total cost.

In general, the development of such innovative, sustainable, and cost-effective method with high yield and purity of lactic acid is still on the horizon. The most promising method for lactic acid production from lignocellulosic feedstocks seems to be pretreatment with DES, followed by SSF (or SSCF) in fed-batch or continuous mode.
